# A Draft Genome Assembly of *Argania spinosa* Provides Insights into Lipid Metabolism-Associated Genes and Repeat-Rich Genome Architecture

**DOI:** 10.3390/plants15192945

**Published:** 2026-09-26

**Authors:** My Abdelmajid Kassem, Naoufal Lakhssassi, Dounya Knizia, Abdelhamid El Mousadik, Khalid Meksem

**Affiliations:** 1 Plant Genomics and Bioinformatics Lab, Department of Biological and Forensic Sciences, Fayetteville State University, Fayetteville, NC 28301, USA; mkassem@uncfsu.edu; 2 Department of Biological Sciences, Hampton University, Hampton, VA 23668, USA; naoufal.lakhssassi@hamptonu.edu; 3 School of Agricultural Sciences, Southern Illinois University, Carbondale, IL 62901, USA; dounya.knizia@siu.edu; 4 Laboratory of Biotechnology and Valorization of Natural Resources, Department of Biology, University Ibn Zohr, Agadir 80000, Morocco; a.elmousadik@uiz.ac.ma

**Keywords:** *Argania spinosa*, de novo assembly, lipid biosynthesis, argan oil, gene ontology, comparative genomics, MEGAHIT, protein-supported annotation, short-read sequencing

## Abstract

*Argania spinosa* (L.) Skeels is an ecologically and economically important Moroccan tree valued for its oil-rich seeds and adaptation to arid environments. Here, we generated and characterized a genotype-specific draft genome assembly of Z-AL-IV-13 using approximately 82.7 GB of Illumina sequencing data. The final contamination-screened assembly comprised 584.21 Mb across 139,591 sequences, with an N50 of 6.84 kb. BUSCO analysis identified 59.1% complete, 25.4% fragmented, and 15.6% missing conserved eudicot orthologs, while independent k-mer analyses indicated incomplete genome representation and substantial repetitive sequence. Comparative whole-genome alignments showed that 95.34–97.60% of the represented Z-AL-IV-13 assembly sequence aligned to five published *A. spinosa* assemblies, indicating high sequence correspondence among represented regions. Protein-supported annotation identified 44,777 transcript models, of which 37,507 (83.77%) produced significant Swiss-Prot matches. RepeatMasker identified 37.73% of the assembly as repetitive, predominantly unclassified interspersed repeats. Targeted homology analyses identified assembly loci associated with fatty-acid and triacylglycerol metabolism, including ACCase components, KAS, SACPD, FAD2, GPAT, LPAT/LPAAT, DGAT, PDAT, and acyl-ACP thioesterase families. These sequence-based assignments identify candidate lipid-metabolism-associated homologs but do not establish pathway completeness, biochemical function, or definitive gene-copy numbers. Overall, Z-AL-IV-13 provides an independently generated, genotype-specific draft genomic resource that complements chromosome-scale *A. spinosa* references while retaining the limitations associated with assembly fragmentation and incomplete gene-space recovery.

## 1. Introduction

The argan tree [*Argania spinosa* (L.) Skeels], an endemic xerophytic species of Morocco, represents a keystone component of arid and semi-arid ecosystems in North Africa. Its natural distribution spans approximately 830,000–900,000 hectares, primarily across the Souss Valley in southwestern Morocco, with additional populations extending toward northern and eastern regions [1,2]. Recognized by UNESCO as a biosphere reserve, the argan ecosystem plays a critical role in biodiversity conservation, desertification prevention, and stabilization of fragile landscapes. Owing to its remarkable tolerance to drought, calcareous substrates, and nutrient-poor soils, *A. spinosa* is also of interest for investigating plant adaptation to abiotic stress and sustainable agriculture under climate change conditions [3].

Ecologically, *A. spinosa* contributes substantially to agroforestry systems by promoting soil conservation, water retention, and habitat stability. However, anthropogenic pressures, including overgrazing, land-use change, and increasing climatic variability, have reduced argan forest density from historical estimates of 150–250 trees per hectare to fewer than 30 in some regions [4,5]. Reduced natural regeneration and progressive ecosystem degradation have intensified the need for effective conservation and restoration strategies. Although propagation approaches such as in vitro culture and enhanced germination protocols have shown partial success, large-scale restoration remains challenging [3,6]. Consequently, understanding the genetic basis of adaptation, reproduction, and stress tolerance in *A. spinosa* is of ecological and agricultural importance.

Beyond its ecological significance, *A. spinosa* has substantial socioeconomic value through the production of argan oil, a high-value product used in culinary, cosmetic, and pharmaceutical applications. Traditional extraction methods remain labor-intensive, often requiring 10–12 h to produce only 1–2 L of oil [4,7], while traditional production continues to support local livelihoods, particularly among Amazigh communities in southwestern Morocco. Argan oil is characterized by a high proportion of unsaturated fatty acids, particularly oleic and linoleic acids, which together comprise approximately 80% of total fatty acids [4]. It also contains tocopherols, sterols such as spinasterol and schottenol, polyphenols, and triterpenes [8,9]. Despite the economic and nutritional importance of argan oil, the genetic and molecular determinants underlying its fatty acid composition and storage-lipid metabolism remain incompletely characterized.

Plant oil biosynthesis involves coordinated fatty acid synthesis and triacylglycerol (TAG) assembly across plastids and the endoplasmic reticulum. Plastid fatty acid synthesis involves acetyl-CoA carboxylase and the fatty acid synthase system, while enzymes such as stearoyl-ACP desaturase contribute to unsaturated fatty acid formation [10,11,12,13]. Further fatty acid modification and glycerolipid and TAG metabolism involve enzymes associated with the endoplasmic reticulum and other cellular compartments, including fatty acid desaturases and components of the Kennedy and related pathways such as GPAT, LPAAT, PAP, and DGAT [11,14,15,16,17,18,19,20,21,22]. Variation in these and related lipid-metabolism genes can influence fatty acid composition and storage-lipid accumulation across plant species [11,14]. Characterization of their homologs in *A. spinosa* therefore provides candidate sequences for investigating argan lipid metabolism, although sequence homology alone does not establish biochemical function, pathway completeness, or specific contributions to argan oil composition.

Cytogenetic studies indicate that *A. spinosa* is diploid but that reported chromosome numbers vary among genotypes [23]. Early karyological studies reported 2n = 20 chromosomes (n = 10), whereas more recent analyses identified chromosome numbers of 2n = 20, 22, and 24 among Moroccan argan genotypes [23]. Recent near-telomere-to-telomere phased genome assemblies resolved 11 pseudochromosomes per haplotype, consistent with a 2n = 22 karyotype [24]. Collectively, these observations indicate cytogenetic variation among reported *A. spinosa* materials and provide additional motivation for genomic characterization across genotypes.

Advances in plant genomics have transformed understanding of genome structure, functional diversity, and evolutionary dynamics across diverse taxa [25,26,27]. Nevertheless, plant genome assembly remains technically challenging because of repetitive sequence, heterozygosity, and genome complexity [28,29,30,31]. These difficulties are particularly relevant to short-read assemblies, in which repetitive regions and alternative haplotypes may remain unresolved and gene models may consequently be fragmented or incomplete.

Recent chromosome-scale and phased genomic resources have substantially advanced the genomic characterization of *A. spinosa* [24,32]. These assemblies provide highly contiguous structural references and establish a genomic framework that cannot be matched in contiguity by the present short-read draft. Accordingly, the Z-AL-IV-13 assembly is not intended as an alternative chromosome-scale reference. Its complementary value lies in providing an independently generated genotype-specific sequence resource for assessing sequence correspondence with published assemblies and for targeted characterization of genomic regions and genes of biological interest.

Plant genome evolution is frequently shaped by whole-genome duplication, segmental duplication, and chromosomal rearrangements, which can contribute to gene-family diversification and metabolic innovation [33]. Comparative genomic studies in plant species have demonstrated the influence of ancestral polyploidization on genome organization and functional diversification. Investigation of such evolutionary processes in *A. spinosa* will ultimately benefit from comparisons across chromosome-scale and genotype-specific genomic resources, although the fragmented nature of short-read assemblies limits inference concerning large-scale structural evolution.

Here, we report a high-coverage Illumina-based draft genome assembly of the Z-AL-IV-13 *A. spinosa* genotype. We assess its completeness using BUSCO and independent k-mer analyses, compare represented sequences with recently published *A. spinosa* reference genomes, characterize repetitive sequence, and evaluate protein-supported gene models. A complementary functional objective is the targeted identification and comparative characterization of candidate homologs associated with fatty acid and triacylglycerol metabolism. Thus, the study is intended neither to establish an alternative chromosome-scale reference nor to demonstrate complete reconstruction of the argan genome or lipid-biosynthesis pathways. Rather, it provides an independently generated genotype-specific draft resource for comparative sequence analysis and targeted investigation of lipid-metabolism-associated genes.

## 2. Results

### 2.1. Genome Assembly and Quality Assessment

De novo assembly of the *A. spinosa* Z-AL-IV-13 genome followed by quality filtering and NCBI contamination screening yielded a final assembly of 584.21 Mb distributed across 139,591 sequences. The assembly had an N50 of 6.84 kb, a maximum sequence length of 154.24 kb, and an overall GC content of 32.35%, reflecting the fragmented nature of this Illumina short-read assembly (Table 1).

Genome completeness was assessed using BUSCO v6.0.0 with the eudicots_odb10 lineage dataset (n = 2326). Using Miniprot v0.13-r248 as the gene predictor, 59.1% of BUSCOs were complete (51.4% single-copy and 7.7% duplicated), 25.4% were fragmented, and 15.6% were missing. An independent BUSCO analysis using MetaEuk v7-bba0d80 produced a closely similar profile, with 58.7% complete (50.2% single-copy and 8.6% duplicated), 24.4% fragmented, and 16.9% missing BUSCOs. The concordance between predictors indicates that the BUSCO profile is not primarily attributable to the choice of gene-prediction algorithm. Rather, the substantial fragmented and missing fractions indicate that conserved gene-space recovery remains incomplete in this short-read assembly.

Independent k-mer analyses further quantified genome representation and assembly complexity. GenomeScope2 analysis of the pooled Illumina reads estimated a haploid genome size of 783–789 Mb using 21-mers and 795–801 Mb using 31-mers, demonstrating consistency across k-mer lengths. Estimated heterozygosity was approximately 1.6% at k = 21 and 1.4% at k = 31. The k-mer-based genome-size estimates therefore exceed the 584.21-Mb assembled span, independently indicating incomplete representation of the underlying genome. GenomeScope2 additionally estimated a substantial repetitive component, accounting for approximately 50–55% of the predicted haploid genome, consistent with the difficulty of resolving repetitive sequence using short reads.

To assess sequence representation independently of assembly span, solid read-derived 21-mers were compared with the final assembly. Using a minimum read abundance of 20, the assembly recovered 455,068,247 of 573,728,301 solid 21-mers (79.32%). Sensitivity analyses using minimum abundance thresholds of 10 and 30 yielded recovery values of 74.15% and 81.34%, respectively. Spectra-cn analysis showed that absent solid 21-mers (CN0) were concentrated near a read multiplicity of approximately 42×, corresponding closely to the heterozygous k-mer peak, whereas once-represented k-mers (CN1) showed their strongest peak near approximately 95×, corresponding to the homozygous-depth peak. This distribution is consistent with alternative allelic or haplotypic variation contributing to the unrepresented k-mer fraction. However, CN1 k-mers were also observed at heterozygous-depth coverage, and the k-mer-based genome-size estimates exceed the assembled span. Consequently, absent k-mers cannot be attributed solely to heterozygosity and likely also include sequence that was not recovered or adequately represented in the fragmented assembly.

The structural characteristics of the assembly are illustrated in Figure 1. The sequence-length distribution was strongly right-skewed, with numerous short sequences and progressively fewer long sequences (Figure 1A), consistent with the low N50 and fragmented structure of the assembly. The cumulative assembly profile showed that longer sequences contributed disproportionately to the total assembled genome span (Figure 1B). GC-content analysis revealed a predominantly unimodal distribution centered at approximately 32–35% (Figure 1C). A small subset of sequences occurred outside the predominant GC-content range, particularly among shorter sequences (Figure S1). These compositionally atypical sequences were subsequently evaluated as part of the contamination-screening workflow described below.

### 2.2. GC-Content Screening and Contamination Assessment

Preliminary GC-content screening identified 3669 sequences outside the 25–45% GC-content interval, representing 6.21 Mb (1.06%) of the pre-screen assembly (Table 2). Most GC-content outliers were short sequences; 39 exceeded 5 kb and collectively represented approximately 270.8 kb. Because atypical GC content alone does not constitute evidence of contamination, these sequences were treated as candidates for further evaluation rather than automatically excluded from the assembly.

The assembly was subsequently evaluated using the NCBI contamination-screening workflow. Of the 141,708 sequences (587.71 Mb) in the pre-screen assembly, 2151 sequences totaling 3,498,876 bp were flagged by NCBI for whole-sequence exclusion, corresponding to 0.595% of the assembly. Among the flagged sequences, the largest taxonomic category was fungi, with 1155 sequences totaling 1,827,672 bp, followed by insects (430 sequences; 774,277 bp) and bacteria (142 sequences; 223,265 bp), with smaller numbers assigned to other categories. In addition, 54 sequences were flagged for trimming or splitting.

Application of the NCBI screening recommendations reduced the assembly by 3,505,780 bp (0.597%), yielding the final contamination-screened assembly of 584,208,796 bp distributed across 139,591 sequences. The difference between the 3,498,876 bp assigned to whole-sequence exclusions and the total 3,505,780-bp reduction reflects the additional sequence removed during trimming and splitting operations. This final contamination-screened assembly was used for all revised downstream assembly-quality, repeat, annotation, and comparative genomic analyses. The contamination-screening and assembly-refinement workflow is summarized in Figure 2.

### 2.3. Repeat Landscape Analysis

De novo repeat discovery and masking analyses revealed a substantial repetitive component within the *A. spinosa* draft assembly. RepeatMasker analysis of the final contamination-screened assembly identified 220.44 Mb of repetitive sequence, corresponding to 37.73% of the 584.21-Mb assembly (Figure 3; Table 3). Unclassified interspersed repeats represented the dominant category, accounting for 204.34 Mb (34.98%), whereas simple repeats accounted for 16.10 Mb (2.76%) of the assembly. Because repeat identification used an assembly-derived de novo repeat consensus library and most interspersed repeat matches could not be assigned to established transposable-element classes, the absence of classified retroelements or DNA transposons in the RepeatMasker output should not be interpreted as evidence that these elements are biologically absent from the *A. spinosa* genome.

Independent GenomeScope2 analysis of the complete Illumina read dataset estimated a larger repetitive component, corresponding to approximately 50–55% of the predicted haploid genome. The higher k-mer-based estimate relative to the assembly-based RepeatMasker estimate is consistent with repetitive sequence that may be incompletely represented in the fragmented short-read assembly or insufficiently classified by the assembly-derived repeat consensus library. Accordingly, the 37.73% RepeatMasker estimate should be interpreted as a conservative estimate of recognizable repetitive sequence represented in the draft assembly, rather than as a definitive estimate of total *A. spinosa* genome repetitiveness.

The substantial repetitive fraction observed in the assembly is consistent with the repeat-rich architecture characteristic of many plant genomes and likely contributed to the fragmented contiguity observed in the short-read assembly. Nevertheless, despite the fragmented nature of the assembly, sufficient protein-coding regions were retained to support protein-supported annotation and reconstruction of biologically relevant metabolic pathways, including lipid biosynthesis.

### 2.4. Comparative Genomic Analysis

Whole-genome alignment of the Z-AL-IV-13 draft against published *A. spinosa* assemblies showed that a high proportion of sequence represented in the draft could be aligned to independently generated argan references. Based on merged non-overlapping query intervals, alignment coverage ranged from 95.34% to 97.60% of the final Z-AL-IV-13 assembly across the five comparisons (Figure 4; Table 4). These percentages quantify the proportion of represented Z-AL-IV-13 sequence aligning to each reference and should therefore be interpreted as evidence of strong sequence correspondence rather than as estimates of genome completeness or recovery.

The highest alignment coverage was observed against the Taguerte25 reference genome (97.60%), followed by NR207 haplotype 2 (97.40%), NR207 hap1_alt1 (97.34%), and NR207 haplotype 1 (96.96%). Alignment to ASM326024v2 yielded a slightly lower coverage of 95.34%. Mean MAPQ values calculated from primary alignments were consistently high across comparisons, ranging from 49.60 to 51.46, providing additional support for the reliability of the mapped sequence correspondence. Together, these results indicate strong similarity between the sequence represented in the Z-AL-IV-13 draft and published *A. spinosa* assemblies despite the fragmentation and incomplete genome representation of the present short-read assembly.

Genome-wide Mash analyses provided an independent, alignment-free assessment of sequence similarity among the *A. spinosa* assemblies (Figure 5; Supplementary Table S1). Pairwise Mash distances across all six assemblies ranged from 0.0056 to 0.0174. Z-AL-IV-13 exhibited Mash distances of 0.0147–0.0174 relative to the five published references, consistent with the high sequence correspondence observed in the whole-genome alignment analyses.

Collectively, the alignment and Mash analyses show that sequences represented in the Z-AL-IV-13 draft correspond closely to independently generated *A. spinosa* genomic resources. These results support the comparative utility of the represented sequence but do not imply completeness of the Z-AL-IV-13 genome assembly.

### 2.5. Protein-Supported Genome Annotation

Protein-supported annotation of the final contamination-screened assembly using the Arabidopsis thaliana TAIR10 reference proteome identified 44,777 transcript models distributed across 14,063 assembly sequences (Figure 6; Table 5). A total of 172,399 coding sequence (CDS) features were identified. These models represent genomic regions supported by similarity to A. thaliana proteins and should therefore be considered homology-supported predictions rather than a comprehensive annotation of the *A. spinosa* gene repertoire.

Protein-supported transcript models had a mean span of 3068.35 bp, while individual CDS features averaged 284.70 bp in length. Each transcript contained an average of 3.85 CDS features. Given the fragmented structure and incomplete gene-space recovery of the Z-AL-IV-13 assembly, some models may represent partial or fragmented genes, and these structural statistics should not be interpreted as evidence of complete gene models.

Subsequent DIAMOND similarity searches against Swiss-Prot identified significant matches for 37,507 of the 44,777 protein-supported models (83.77%; Figure 6). Among these matches, 27,116 (72.30%) had A. thaliana as the best-matching Swiss-Prot species. This predominance should be interpreted in the context of the annotation strategy, because TAIR10 proteins were used to generate the initial protein-supported models and therefore the species distribution of subsequent best matches does not constitute independent evidence of evolutionary proximity. Gene Ontology (GO) mapping provided at least one current GO assignment for 36,273 models with Swiss-Prot support. Together, these results provide functional context for many of the homology-supported models while remaining consistent with the incomplete conserved gene-space recovery indicated by BUSCO analyses.

### 2.6. Functional Annotation

Of the 44,777 protein-supported transcript models identified in the final contamination-screened *A. spinosa* assembly, 37,507 (83.77%) exhibited significant sequence similarity to proteins in the curated UniProt Swiss-Prot database (Figure 6). Among these matches, 27,116 (72.30%) had Arabidopsis thaliana as the best-matching Swiss-Prot species, while additional best matches were associated with other plant species, including *Oryza sativa*, *Glycine max*, *Vitis vinifera*, *Solanum lycopersicum*, and *Pisum sativum*. The predominance of *A. thaliana* matches should be interpreted in the context of the annotation strategy, because TAIR10 proteins were used to generate the initial protein-supported models and therefore this distribution does not constitute independent evidence of evolutionary proximity.

Gene Ontology mapping provided at least one current GO assignment for 36,273 of the protein-supported models with Swiss-Prot matches. Among these models, 31,407 were associated with Biological Process terms, 31,833 with Molecular Function terms, and 33,013 with Cellular Component terms; these categories overlap because individual models may be assigned to more than one GO namespace. Frequently represented Biological Process terms included regulation of DNA-templated transcription, signal transduction, protein ubiquitination, and responses to abscisic acid, defense, salt, water deprivation, and cold. Prominent Molecular Function assignments included ATP binding, metal-ion binding, DNA-binding transcription-factor activity, zinc-ion binding, DNA binding, and protein kinase activity, while frequently represented Cellular Component terms included the nucleus, cytoplasm, plasma membrane, cytosol, chloroplast, and mitochondrion.

These similarity- and ontology-based assignments provided functional context for the homology-supported models and supported subsequent targeted characterization of candidate homologs associated with fatty-acid and triacylglycerol metabolism. However, the annotations are based primarily on sequence homology and should not be interpreted as experimental confirmation of biochemical function or as evidence of complete gene-space recovery.

### 2.7. Targeted Identification of Lipid Metabolism-Associated Homologs

Targeted homology analysis identified protein-supported assembly loci associated with multiple components of fatty-acid and triacylglycerol metabolism in the Z-AL-IV-13 draft assembly (Figure 7; Table 6). Homology-supported loci included components of fatty-acid synthesis, such as acetyl-CoA carboxylase components, acyl carrier proteins, malonyl-CoA:ACP transacylase, ketoacyl-ACP synthases (KASI, KASII, and KASIII), 3-oxoacyl-ACP reductase, 3-hydroxyacyl-ACP dehydratase, enoyl-ACP reductase, stearoyl-ACP desaturase, and acyl-ACP thioesterases (FATA, FATB, and TE3). Homologs associated with downstream glycerolipid, and triacylglycerol metabolism included GPAT, LPAT/LPAAT, PAH, DGAT, PDAT, LPCAT, and PDCT/ROD1 families.

Two distinct FAD2-like loci and three omega-3-fatty-acid-desaturase-like loci were identified. Comparative sequence analysis favored chloroplast-associated FAD7/FAD8 assignments for the omega-3-desaturase-like loci, although one locus was less confidently resolved. A distinct endoplasmic-reticulum-associated FAD3 locus was not confidently resolved in the present assembly and was therefore not included as a supported locus in Table 6. Because the assembly is fragmented and the annotation is based primarily on protein homology, these loci represent candidate homologs supported by the assembled sequence and should not be interpreted as evidence of pathway completeness or definitive gene-family copy number.

Candidate homologs representing multiple stages of fatty-acid synthesis, modification, and triacylglycerol metabolism were recovered among the protein-supported models. These included homologs associated with acetyl-CoA carboxylase, KAS, SACPD, FAD2, GPAT, LPAT/LPAAT, PAH, DGAT, PDAT, LPCAT, PDCT/ROD1, and acyl-ACP thioesterase families. Sequence-similarity evidence supports classification of these models within conserved plant lipid-metabolism-associated protein families; however, the fragmented assembly and homology-based annotation limit inference of complete gene structures and family membership for some loci.

Because the assembly exhibits incomplete gene-space recovery, the observed numbers of candidate homologs should not be interpreted as definitive biological gene-family copy numbers, and apparent absence of additional family members cannot be interpreted as biological gene loss. Furthermore, sequence similarity alone does not establish gene expression, enzymatic activity, or a specific contribution to argan oil composition. The identified sequences therefore represent candidates for subsequent functional characterization rather than evidence that the complete lipid-biosynthesis pathway has been reconstructed.

Phylogenetic analysis provided an additional line of evidence for classification of selected *A. spinosa* fatty acid desaturase candidates (Figure 8). The analysis supported classification of two candidates as FAD2-like sequences and favored FAD7/FAD8-like classification for three omega-3-desaturase-like candidates, although one of the latter was less confidently resolved. A distinct ER-associated FAD3 candidate was not confidently resolved. These phylogenetic relationships support sequence-based family classification but do not establish the biochemical functions of the corresponding *A. spinosa* proteins.

### 2.8. Gene Ontology Functional Categories

Gene Ontology (GO) mapping provided functional assignments for 36,273 protein-supported models with Swiss-Prot matches (Figure 9; Supplementary Table S3). Among these models, 31,407 were associated with Biological Process terms, 31,833 with Molecular Function terms, and 33,013 with Cellular Component terms. These counts are non-exclusive because individual protein models may be associated with terms from multiple GO namespaces.

Frequently represented Cellular Component terms included nucleus (GO:0005634), cytoplasm (GO:0005737), plasma membrane (GO:0005886), cytosol (GO:0005829), chloroplast (GO:0009507), and mitochondrion (GO:0005739). Frequently represented Molecular Function terms included ATP binding (GO:0005524), metal ion binding (GO:0046872), DNA-binding transcription factor activity, zinc ion binding (GO:0008270), DNA binding (GO:0003677), and protein serine kinase activity. Among Biological Process annotations, frequently represented terms included regulation of DNA-templated transcription, signal transduction, protein ubiquitination, response to abscisic acid, defense response, response to salt stress, response to water deprivation, and response to cold (Supplementary Table S3).

Overall, the GO mapping indicates that the homology-supported models represented in the Z-AL-IV-13 assembly span diverse cellular, regulatory, signaling, stress-response, and metabolic functions. These assignments provide functional context for the annotated protein set and complement the targeted characterization of lipid-metabolism-associated homologs. However, because GO assignments were derived from Swiss-Prot homolog associations and the draft assembly shows incomplete gene-space recovery, the observed functional representation should be interpreted as a description of the annotated models rather than as a comprehensive characterization of the complete *A. spinosa* functional repertoire.

## 3. Discussion

The availability of chromosome-scale and phased *A. spinosa* genomes necessarily defines the scope of the present draft assembly. With an N50 of 6.84 kb, Z-AL-IV-13 is substantially more fragmented than these recent references and is not intended to replace them as a structural genomic resource. Its value instead lies in providing an independently sequenced and assembled genotype-specific dataset that can be compared with those references and used for targeted characterization of lipid-metabolism-associated genes. The strong correspondence of the assembled Z-AL-IV-13 sequence to independently generated argan genomes supports conservation of many represented regions, whereas BUSCO and k-mer analyses simultaneously demonstrate substantial sequence and gene-space incompleteness. Within these limitations, protein-supported annotation and targeted analyses identified candidate homologs associated with fatty-acid and triacylglycerol metabolism, providing a complementary functional component to the comparative genomic analysis. Thus, the contribution of Z-AL-IV-13 is not as an alternative to chromosome-scale reference genomes, but as an independent genotype-specific resource for comparative sequence analysis and targeted investigation of lipid-metabolism-associated genes.

### 3.1. Challenges of Short-Read Genome Assembly in Plants

The assembly of plant genomes remains technically challenging because of extensive repetitive DNA, structural complexity, and varying levels of heterozygosity, all of which can reduce assembly contiguity when only short-read sequencing data are available [28,35,36]. These challenges are particularly relevant to *A. spinosa*, for which the present k-mer analyses indicate substantial genome complexity and heterozygosity.

GenomeScope2 analysis provided additional context for the fragmented Z-AL-IV-13 assembly. Estimates obtained using two k-mer lengths were consistent, with a predicted haploid genome size of 783–789 Mb at k = 21 and 795–801 Mb at k = 31, and heterozygosity estimates of approximately 1.6% and 1.4%, respectively. In comparison, the final contamination-screened assembly spans 584.21 Mb, corresponding to approximately three-quarters of the predicted haploid genome size. GenomeScope2 further estimated that repetitive sequence accounts for approximately 50–55% of the haploid genome. Together, the substantial repetitive component and relatively high heterozygosity provide plausible contributors to the difficulty of reconstructing the genome from short Illumina reads, while the difference between estimated genome size and assembled sequence demonstrates that the draft remains incomplete.

Read-derived k-mer recovery independently supported this interpretation. At the primary minimum-abundance threshold of 20, the assembly contained 79.32% of solid read-derived 21-mers, with recovery estimates of 74.15% and 81.34% at minimum-abundance thresholds of 10 and 30, respectively. Spectra-cn analysis further showed that absent solid 21-mers were concentrated near the heterozygous-depth peak (~42×), indicating that alternative allelic or haplotypic variation contributes to the unrepresented k-mer fraction. However, because once-represented assembly k-mers were also observed at heterozygous-depth coverage and the assembly spans only approximately three-quarters of the k-mer-estimated haploid genome, heterozygosity alone cannot account for the missing sequence. Repetitive sequence, unresolved allelic variation, and other regions not recovered by the short-read assembly likely contribute collectively to the observed incompleteness.

These results clarify both the utility and limitations of the Z-AL-IV-13 assembly. The draft contains sufficient represented sequence to support comparative sequence analyses and targeted identification of candidate genes, but it does not provide a complete representation of the *A. spinosa* genome. Chromosome-scale assemblies therefore remain essential for analyses requiring long-range structural organization, repetitive-region resolution, and haplotype architecture, whereas the present dataset is most appropriately used as a complementary genotype-specific resource for comparative and targeted functional analyses.

### 3.2. Contamination Screening and Assembly Refinement

Contamination screening represents an essential quality-control step in modern genome assembly workflows, particularly for plant genomes where environmental microorganisms and endophytic fungi may co-purify with host DNA during sample preparation [37]. In the present study, preliminary GC-content profiling and sequence-similarity searches were used to identify compositionally atypical sequences; however, GC content alone was not considered sufficient evidence for contamination because authentic plant sequences may also occur outside the predominant genomic GC range. The assembly was therefore subjected to an additional NCBI contamination screen, and the final assembly was generated by applying the resulting sequence-exclusion and trimming recommendations.

The pre-screen assembly contained 141,708 sequences totaling 587.71 Mb. NCBI contamination screening identified 2151 sequences, totaling 3.50 Mb (0.595% of the pre-screen assembly), for whole-sequence exclusion and identified 54 additional sequences requiring trimming or splitting. The excluded sequences included matches assigned predominantly to fungal, insect, and prokaryotic sources, with ascomycete-like sequences representing the largest category among the whole-sequence exclusions. Application of the complete NCBI screening recommendations, including removal of flagged sequences and trimming or splitting of affected sequences, reduced the assembly by approximately 3.51 Mb (0.597%) and produced a final contamination-screened assembly of 584.21 Mb distributed across 139,591 sequences.

The small proportion of sequence removed indicates that contamination did not account for the overall fragmentation or reduced genome representation of the Z-AL-IV-13 assembly. This conclusion is further supported by the observation that BUSCO completeness was unchanged between the originally analyzed assembly and the final NCBI-screened assembly, with both yielding 59.1% complete eudicot BUSCOs under the same Miniprot-based analysis. Thus, contamination screening improved confidence that downstream comparative and annotation analyses were based predominantly on target genomic sequence, while the principal limitations of the final assembly—fragmentation and incomplete genome recovery—remained evident after screening.

### 3.3. Comparative Genomics and Genome Recovery

Comparative genomic analyses provided an independent assessment of the correspondence between sequences represented in the Z-AL-IV-13 assembly and previously published *A. spinosa* genomes. Despite its substantially lower contiguity relative to the chromosome-scale references, whole-genome alignments showed that 95.34–97.60% of the final Z-AL-IV-13 assembly sequence aligned to five independently generated reference assemblies. Mean MAPQ values for primary alignments ranged from 49.60 to 51.46, providing additional support for the reliability of the mapped sequence correspondence. These results indicate that sequences represented in the draft correspond closely to independently assembled *A. spinosa* genomic sequences [32,38,39].

Mash analyses provided a complementary alignment-independent assessment of overall sequence similarity. Pairwise Mash distances across all six assemblies ranged from 0.0056 to 0.0174, while distances between Z-AL-IV-13 and the five published references ranged from 0.0147 to 0.0174. The consistency between minimap2-based alignments and Mash analyses supports strong overall sequence similarity among the represented genomic datasets despite differences in sequencing technologies, assembly strategies, and haplotype representation.

Importantly, high reference-alignment coverage should not be interpreted as equivalent to high genome completeness. The alignment percentages describe the correspondence of sequence present in Z-AL-IV-13 to published *A. spinosa* references, whereas the independent BUSCO and k-mer analyses assess different aspects of genome and gene-space representation. BUSCO recovered approximately 59% complete conserved eudicot orthologs, while GenomeScope2 estimated a haploid genome size of approximately 0.79–0.80 Gb compared with the 584.21-Mb assembled sequence. These independent analyses therefore indicate substantial residual incompleteness in the Z-AL-IV-13 draft. Comparative alignment and completeness analyses should consequently be viewed as complementary: the former shows that most sequence present in the assembly corresponds closely to independently generated argan genomic sequence, whereas the latter indicates incomplete representation of the underlying genome and conserved gene space.

Accordingly, the principal comparative value of Z-AL-IV-13 lies in providing an independently generated genotype-specific sequence dataset rather than an alternative structural reference genome. Its strong sequence correspondence with published *A. spinosa* assemblies supports its use for comparative analysis of represented regions and targeted investigation of candidate genes, including homologs associated with lipid metabolism. Analyses requiring chromosome-scale continuity, more comprehensive gene-space representation, or long-range structural resolution are better addressed using the recently available chromosome-scale resources [40,41].

### 3.4. Repetitive Elements and Genome Architecture

Repetitive DNA constitutes a major structural component of plant genomes and is an important factor limiting the contiguity of short-read genome assemblies [28,36]. RepeatMasker analysis of the final contamination-screened Z-AL-IV-13 assembly identified 37.73% of the assembled sequence as repetitive. Most of this sequence (34.98% of the assembly) consisted of unclassified interspersed repeats, while 2.76% was identified as simple repeats. No major retroelement or DNA-transposon classes were resolved using the assembly-derived de novo repeat consensus library. The absence of these classified categories should not be interpreted as evidence that such elements are biologically absent from the *A. spinosa* genome. Rather, the RepeatMasker value represents a conservative estimate of repetitive sequence recognizable under the present assembly and library conditions.

Independent k-mer modeling supports a larger repetitive component. GenomeScope2 estimated repetitive sequence at approximately 50–55% of the predicted haploid genome, exceeding the 37.73% identified by RepeatMasker in the assembled sequence. This discrepancy is consistent with repetitive sequence being incompletely represented, collapsed, or insufficiently classified in the fragmented short-read assembly. Limited taxonomic representation of Sapotaceae repeats in available resources may additionally constrain repeat classification, although the present analysis does not distinguish this effect from limitations associated with assembly fragmentation or the de novo consensus library.

The substantial repetitive component is also consistent with repeat-associated difficulties in reconstructing the Z-AL-IV-13 genome from short reads, particularly across long or highly similar repetitive regions and other complex genomic structures [35,42]. Nevertheless, the present analyses do not quantify the specific contribution of repetitive DNA to individual assembly gaps or missing genomic regions. Accordingly, the RepeatMasker results are most appropriately interpreted as a conservative characterization of recognizable repetitive sequence represented in the draft rather than a comprehensive description of *A. spinosa* repeat composition. More contiguous assemblies and improved taxonomically representative repeat libraries will be required to resolve the abundance, diversity, and genomic organization of repetitive elements in greater detail.

### 3.5. Functional Annotation and Protein-Coding Gene Representation

Protein-supported annotation identified 44,777 transcript models and 172,399 coding sequence (CDS) features within the final contamination-screened *A. spinosa* Z-AL-IV-13 assembly, with protein-supported models distributed across 14,063 assembly sequences. These models were generated by mapping Arabidopsis thaliana TAIR10 proteins to the draft genome and therefore represent homology-supported predictions rather than a comprehensive de novo annotation of the *A. spinosa* gene repertoire. The mean transcript span was 3068.35 bp, while the mean CDS feature length was 284.70 bp, with an average of 3.85 CDS features per transcript. Given the fragmented nature of the underlying assembly, some predicted genes are likely represented by partial or fragmented models.

Functional similarity searches identified significant Swiss-Prot homologs for 37,507 of the 44,777 protein-supported models (83.77%). Of these Swiss-Prot-supported models, 27,116 (72.30%) had their best match to A. thaliana. This predominance should be interpreted in the context of the TAIR10 protein set used to generate the initial protein-supported models and therefore does not constitute independent evidence of unusually close evolutionary affinity to Arabidopsis. Gene Ontology terms were associated with 36,273 models, representing diverse biological processes, molecular functions, and cellular components. These results indicate that numerous conserved plant protein families and functional categories are represented among sequences recovered in the draft assembly, but they do not imply comprehensive recovery of the *A. spinosa* gene repertoire.

This distinction is consistent with the BUSCO analyses. Miniprot-based BUSCO analysis recovered 59.1% complete, 25.4% fragmented, and 15.6% missing conserved eudicot orthologs, while the independent MetaEuk analysis produced a similar profile, with 58.7% complete, 24.4% fragmented, and 16.9% missing orthologs. The agreement between these approaches indicates that the observed gene-space incompleteness is not attributable solely to the gene-prediction method used by BUSCO. The relatively high proportion of protein-supported models with Swiss-Prot matches and the lower BUSCO completeness estimates measure different properties: the former assesses detectable homology among models that were recovered, whereas the latter evaluates recovery of an expected conserved ortholog set. Taken together, these results support use of the Z-AL-IV-13 assembly for targeted identification and comparative analysis of represented genes while emphasizing that apparent gene absences, gene-family sizes, and gene-copy numbers should be interpreted cautiously in this fragmented draft.

### 3.6. Lipid Metabolism-Associated Homologs and Biological Relevance

An important functional objective of this study was the targeted identification and characterization of candidate homologs associated with fatty acid biosynthesis and triacylglycerol (TAG) metabolism. These metabolic processes underpin plant seed-oil production and are therefore relevant to the nutritional, ecological, and economic importance of *A. spinosa* [11,14,19,43].

Homology-supported loci were identified for multiple components associated with plastid fatty acid synthesis and downstream glycerolipid and TAG metabolism. These included cytosolic and plastid acetyl-CoA carboxylase components, acyl carrier proteins, malonyl-CoA:ACP transacylase, ketoacyl-ACP synthases (KASI, KASII, and KASIII), 3-oxoacyl-ACP reductase, 3-hydroxyacyl-ACP dehydratase, enoyl-ACP reductase, stearoyl-ACP desaturase (SACPD), fatty acid thioesterases, fatty acid desaturases, GPAT, LPAT/LPAAT, PAH, DGAT, PDAT, LPCAT, and PDCT/ROD1. Their identification indicates that the assembled sequence contains homologs associated with multiple stages of plant fatty acid and TAG metabolism. However, given the fragmentation and incomplete gene-space recovery of the draft genome, these observations do not demonstrate complete reconstruction of the corresponding metabolic pathways.

The fatty acid desaturase candidates illustrate the importance of cautious family-level assignment. Two distinct FAD2-like loci were supported in the assembly, whereas three additional omega-3-desaturase-like loci showed sequence relationships favoring chloroplast-associated FAD7/FAD8 assignments, with one locus less confidently resolved. A distinct endoplasmic-reticulum-associated FAD3 locus was not confidently resolved in the present assembly. This result should not be interpreted as evidence that FAD3 is biologically absent from *A. spinosa*, because incomplete assembly and homology-based annotation may prevent recovery or confident classification of individual family members. FAD2-like sequences remain of particular interest because endoplasmic-reticulum-associated desaturases participate in the formation of polyunsaturated fatty acids in plants [12,13,44]. SACPD-like loci are likewise relevant because stearoyl-ACP desaturases participate in the formation of oleoyl-ACP during plastid fatty acid biosynthesis. Together with the other lipid-metabolism-associated homologs identified here, these loci provide candidates for further investigation of fatty acid and storage-lipid metabolism in *A. spinosa*. Their identification alone, however, does not establish expression, enzymatic activity, or a specific contribution to argan oil composition.

Apparent gene-family sizes should also be interpreted cautiously. Fragmented assemblies and homology-based annotation can generate overlapping, partial, or split protein-supported models, whereas incomplete genome recovery may result in genuine family members not being represented. For this reason, overlapping or fragmented models associated with the same genomic region were consolidated during the locus-level interpretation used for Table 6. The reported values therefore represent homology-supported assembly loci rather than definitive biological gene-copy numbers. More complete genome annotation supported by transcriptomic evidence will be required to refine gene structures, distinguish paralogous loci from assembly or annotation artifacts, and assess expression in oil-producing tissues.

Phylogenetic analysis provided an additional line of evidence for sequence-based classification of selected fatty acid desaturase candidates. Comparison with characterized reference desaturases supported the assignment of two loci as FAD2-like and favored FAD7/FAD8-like classification of the recovered omega-3-desaturase candidates rather than confident assignment of a distinct FAD3 locus. Nevertheless, phylogenetic placement and sequence homology do not by themselves demonstrate enzymatic function. Functional characterization will require complementary evidence such as expression profiling, improved gene models, and biochemical or genetic validation.

Overall, the identification of homology-supported loci associated with multiple stages of fatty acid and TAG metabolism provides a genotype-specific set of candidate sequences for future investigation of lipid biology in *A. spinosa*. These findings complement the comparative genomic characterization of Z-AL-IV-13 but should be interpreted as evidence for the representation of lipid-metabolism-associated homologs in the draft assembly rather than proof of pathway completeness or of their specific roles in determining argan oil composition.

### 3.7. Gene Ontology Functional Landscape

Gene Ontology (GO) mapping of the annotated *A. spinosa* protein models provided functional assignments for 36,273 protein-supported models represented in the Z-AL-IV-13 assembly. Among these models, 31,407 were associated with biological process (BP) terms, 31,833 with molecular function (MF) terms, and 33,013 with cellular component (CC) terms. Because individual models may receive multiple GO terms and assignments across more than one namespace, these categories are non-exclusive and should not be summed.

Frequently represented biological process terms included regulation of DNA-templated transcription, signal transduction, protein ubiquitination, and responses to abscisic acid, salt stress, water deprivation, and cold. Prominent molecular function terms included ATP binding, metal ion binding, DNA-binding transcription factor activity, zinc ion binding, DNA binding, and protein kinase activity. Cellular component assignments were frequently associated with the nucleus, cytoplasm, plasma membrane, cytosol, chloroplast, and mitochondrion. Together, these assignments indicate that the protein-supported models span diverse conserved plant cellular, regulatory, metabolic, and stress-associated functions.

The GO results complement the protein-supported annotation by demonstrating functional representation across multiple categories rather than restriction to the lipid-metabolism-associated candidates examined in greater detail. However, the GO assignments were derived from homology to Swiss-Prot proteins and therefore provide functional annotations rather than experimental evidence of gene activity or functional enrichment. Moreover, because the underlying assembly exhibits substantial fragmentation and incomplete gene-space recovery, the frequency of individual GO terms should not be interpreted as a comprehensive representation of the complete *A. spinosa* functional repertoire.

Overall, GO mapping provides functional context for the protein-supported models represented in the Z-AL-IV-13 draft and supports their use in comparative and targeted gene analyses. These assignments describe the functional diversity of recovered models while remaining subject to the limitations of homology-based annotation, assembly fragmentation, and incomplete gene-space recovery.

### 3.8. Limitations and Future Directions

The principal limitations of the present study arise from the fragmented and incomplete nature of the short-read Z-AL-IV-13 assembly and the reliance on homology-based gene annotation. The final assembly comprises 584.21 Mb, whereas GenomeScope2 estimated a haploid genome size of approximately 0.79–0.80 Gb. BUSCO analyses recovered approximately 59% complete conserved eudicot orthologs, with substantial fragmented and missing fractions. In addition, solid read-derived 21-mer analysis recovered 79.32% of k-mers at a minimum abundance threshold of 20. Together, these independent measures indicate incomplete representation of the underlying genome and conserved gene space. These limitations restrict the resolution of repetitive regions, structural variation, haplotype organization, and chromosome-scale genome architecture and may also result in incomplete, split, or missing gene models. Consequently, apparent gene-family sizes and gene absences should be interpreted cautiously, particularly for the lipid-metabolism-associated candidates examined in this study.

Functional interpretation is subject to additional limitations. The identification of homologs associated with fatty acid and triacylglycerol metabolism establishes the presence of candidate sequences but does not demonstrate pathway completeness, gene expression, enzymatic activity, or specific contributions to argan oil composition. Similarly, protein-similarity and GO assignments provide homology-based functional annotations rather than experimental validation. Transcriptomic evidence from relevant tissues and developmental stages, together with improved structural annotation and biochemical or genetic analyses, will therefore be required to validate candidate gene functions and clarify their roles in *A. spinosa* lipid metabolism.

Future work should focus on integrating long-read sequencing technologies, including PacBio HiFi and Oxford Nanopore sequencing, to improve assembly contiguity, resolve repetitive regions, and characterize haplotype structure more completely [45,46]. Additional scaffolding approaches, such as Hi-C chromatin conformation capture and optical mapping, could facilitate chromosome-scale genome reconstruction and enable more comprehensive analyses of genome organization [47]. Integration of transcriptomic, comparative genomic, and functional genomic datasets would further refine gene models and provide a stronger basis for investigating lipid metabolism, environmental adaptation, stress responses, and genome evolution in *A. spinosa*.

## 4. Materials and Methods

### 4.1. Plant Material, Sequencing, and Read Preprocessing

Genomic DNA was extracted from young leaf tissue of a mature *A. spinosa* individual (genotype Z-AL-IV-13) maintained in the greenhouse facility of the Horticulture Research Center at Southern Illinois University Carbondale. Seeds corresponding to this genotype were originally collected from the Souss Valley region of southwestern Morocco and provided by Prof. Abdelhamid El Mousadik.

Whole-genome sequencing libraries were prepared from genomic DNA and sequenced using the Illumina HiSeq X platform (Illumina, Inc., San Diego, CA, USA), generating approximately 82.7 GB of raw paired-end sequence data. Raw paired-end reads were processed using fastp v0.23. Adapter sequences were detected and removed, and reads were subjected to quality trimming and filtering before genome assembly. Read-quality statistics before and after preprocessing were evaluated using the quality-control reports generated by fastp. The resulting quality-filtered paired-end reads were used for de novo genome assembly and subsequent read-based k-mer analyses.

### 4.2. De Novo Genome Assembly

De novo genome assembly was performed using MEGAHIT v1.2, a de Bruijn graph-based short-read assembler. Assembly was conducted using an iterative series of k-mer sizes of 21, 29, 39, 59, 79, 99, 119, and 141. Unless otherwise specified, MEGAHIT was run using default parameters. Genome assembly was performed on Amazon Web Services Elastic Compute Cloud (AWS EC2) computing instances configured with 128 GB of RAM.

Following assembly, sequences shorter than 1000 bp were initially excluded to reduce the contribution of highly fragmented sequences to subsequent analyses. The resulting assembly was subsequently subjected to contamination screening and refinement as described in Section 4.4. Because the later contamination-screening procedure included sequence trimming and splitting, the final contamination-screened assembly could contain sequences shorter than the initial 1000-bp filtering threshold.

Assembly statistics were calculated for the final contamination-screened assembly, including total assembly size, number of sequences, GC content, N50, N90, L50, L90, maximum sequence length, and sequence-length distributions. The final contamination-screened assembly was used as the reference for all revised downstream assembly-quality, comparative genomic, repeat, annotation, and functional analyses.

### 4.3. Assembly Quality Assessment

Assembly quality was evaluated using complementary structural, conserved gene-space, and k-mer-based metrics. Standard assembly statistics, including total assembly length, number of sequences, N50, N90, L50, L90, maximum sequence length, GC content, and sequence-length distributions, were calculated using QUAST (v5.2) [48].

Conserved gene-space completeness was assessed using BUSCO (v6.0.0) [49] in genome mode with the eudicots_odb10 lineage dataset (n = 2326). The primary analysis used Miniprot [50] for gene prediction, and BUSCO results were classified as complete single-copy, complete duplicated, fragmented, or missing according to the standard BUSCO framework. To evaluate whether the completeness estimate was strongly dependent on the gene-prediction method, an independent BUSCO analysis was performed on the same final contamination-screened assembly using MetaEuk as the gene predictor and the same eudicots_odb10 lineage dataset.

Independent k-mer-based analyses were performed using the complete Illumina read dataset. Read-derived k-mer frequency distributions were generated using KMC (v3.2.4). GenomeScope2 was applied independently to k-mer distributions generated at k = 21 and k = 31 to estimate haploid genome size, heterozygosity, and repetitive sequence content.

Assembly representation was further evaluated by comparing read-derived solid 21-mers with 21-mers present in the final contamination-screened assembly. A minimum read abundance of 20× was used as the primary threshold for defining solid k-mers, based on the observed valley separating the low-frequency, error-associated portion of the k-mer distribution from the principal genomic k-mer peaks. Sensitivity analyses were additionally performed using minimum abundance thresholds of 10× and 30×. Solid k-mer recovery was calculated as the proportion of read-derived solid k-mers detected in the final assembly.

To examine the relationship between read k-mer abundance and representation in the assembly, solid read-derived 21-mers were additionally partitioned according to their copy number in the final assembly as CN0 (absent), CN1, CN2, CN3, or CN4+ (four or more copies). Read-abundance distributions were then examined separately for each assembly copy-number class and compared with the major heterozygous- and homozygous-depth peaks. This analysis was used to characterize patterns of k-mer representation rather than as an independent estimate of genome completeness.

GC-content distributions and sequence-length profiles were additionally examined across the assembly. Statistical summaries and figures were generated using custom Python v3.10.20 scripts with pandas v2.3.3, NumPy v2.2.6, and matplotlib v3.10.9.

### 4.4. Contamination Screening and Assembly Refinement

Potential compositionally atypical sequences were initially identified by examining genome-wide GC-content distributions. Sequences with GC content outside the 25–45% interval were designated as GC-content outliers for further evaluation. Because atypical GC content alone is not sufficient evidence of contamination, these sequences were not automatically removed on the basis of nucleotide composition. The number and total length of GC-content outliers were recorded, and sequences exceeding 5 kb were examined separately to prioritize longer compositionally atypical sequences for sequence-similarity assessment.

Preliminary sequence-similarity screening of selected GC-content outliers was performed using BLASTn v2.16 against the NCBI nucleotide (nt) database. Similarity matches were evaluated with respect to taxonomic assignment, sequence identity, alignment coverage, and statistical significance. This preliminary analysis was used to investigate the possible origins of compositionally atypical sequences rather than to classify all GC-content outliers as contaminants or to define the final set of sequences removed from the assembly.

The assembly was subsequently subjected to the NCBI contamination-screening workflow, which was used to define the final assembly refinement. Sequences flagged by the NCBI screen for whole-sequence exclusion were removed according to the screening recommendations, whereas sequences flagged for partial sequence removal were trimmed or split at the indicated coordinates. The pre-screen assembly contained 141,708 sequences totaling 587,714,576 bp. NCBI screening flagged 2151 sequences totaling 3,498,876 bp for whole-sequence exclusion and 54 sequences for trimming or splitting. Following application of these recommendations, the assembly was reduced by 3,505,780 bp (0.597%), yielding a final contamination-screened assembly of 584,208,796 bp distributed across 139,591 sequences. This final assembly was used as the reference for all revised downstream assembly-quality, comparative genomic, repeat, annotation, and functional analyses.

### 4.5. Comparative Genomic Analysis

The Z-AL-IV-13 *A. spinosa* draft assembly was compared with five publicly available *A. spinosa* genome assemblies: Taguerte25, NR207_hap1, NR207_hap2, NR207_hap1_alt1 [32], and ASM326024v2 [24]. Whole-genome pairwise alignments were performed using minimap2 v2.31-r1302 with the asm5 preset for assembly-to-assembly sequence comparison and eight computational threads.

Alignment outputs were generated in Pairwise Alignment Format (PAF) and processed using custom scripts. To avoid double-counting sequence represented by overlapping alignments, aligned intervals on each Z-AL-IV-13 query sequence were merged before calculating the total number of uniquely aligned query bases. Alignment coverage was calculated as the total length of these merged intervals divided by the total length of the final contamination-screened Z-AL-IV-13 assembly (584,208,796 bp). Accordingly, the reported coverage values quantify the proportion of sequence represented in Z-AL-IV-13 that aligned to each published reference and should not be interpreted as the proportion of each reference genome recovered by the Z-AL-IV-13 assembly. Mean mapping quality (MAPQ) was calculated from primary minimap2 alignments identified by the tp:A:P PAF tag.

Genome-wide sequence similarity among the six assemblies was additionally evaluated using Mash v2.3, which uses MinHash sketching to estimate pairwise genomic distances independently of whole-genome alignment. Pairwise Mash distances were calculated among Z-AL-IV-13 and the five published *A. spinosa* assemblies and visualized as a distance heatmap, providing a complementary alignment-free assessment of overall sequence similarity among the genomic datasets.

### 4.6. Protein-Supported Genome Annotation

Protein-supported genome annotation was performed using miniprot v0.13-r248, a splice-aware protein-to-genome alignment program. Protein sequences from the Arabidopsis thaliana TAIR10 reference proteome [51] were aligned against the final contamination-screened *A. spinosa* Z-AL-IV-13 assembly to identify genomic regions supported by homology to characterized plant proteins and to infer corresponding exon–intron structures. TAIR10 was selected as a well-curated plant reference proteome suitable for identifying conserved protein-coding sequences. Because this approach relies on protein homology to a single reference species, it was used to generate protein-supported gene models rather than a comprehensive de novo annotation of the *A. spinosa* gene repertoire.

Genome-wide protein-to-genome alignments were performed using 16 computational threads within an Amazon Web Services Elastic Compute Cloud (AWS EC2) computing environment. Alignment results were generated in Pairwise Alignment Format (PAF) and GFF3 format. Protein-supported transcript models, coding sequence (CDS) features, and assembly sequences containing predicted models were quantified using custom shell scripts and downstream statistical analyses.

Protein and CDS sequences were subsequently extracted from the miniprot-derived annotations using gffread v0.12.9 [52] after removal of miniprot-specific ##PAF records from the annotation file to ensure compatibility with downstream GFF3 processing. Annotation statistics included the number of protein-supported transcript models, number of CDS features, mean transcript span, mean CDS feature length, mean number of CDS features per transcript, and number of assembly sequences containing protein-supported models. Given the fragmented nature and incomplete conserved gene-space recovery of the assembly, these models were interpreted as homology-supported predictions and were not assumed to represent complete gene structures or the complete *A. spinosa* protein-coding gene repertoire.

### 4.7. Functional Annotation and Gene Ontology Analysis

Predicted protein sequences derived from the protein-supported gene models were functionally annotated using DIAMOND v2.1.16 [53] against the manually curated UniProt Swiss-Prot protein database [54], containing 574,627 protein sequences at the time of analysis. Sequence-similarity searches were performed using DIAMOND blastp with an E-value threshold of 1 × 10^−5^, with a maximum of one target sequence retained per query protein. The highest-scoring Swiss-Prot match was therefore used for downstream functional annotation.

Putative functional descriptions were assigned to the predicted *A. spinosa* proteins on the basis of their retained Swiss-Prot homologs. Annotation statistics, including the number and proportion of predicted proteins with significant Swiss-Prot matches, were subsequently calculated. Because these assignments were based on sequence similarity, they were interpreted as homology-supported functional annotations rather than experimental confirmation of protein function in *A. spinosa*.

Gene Ontology (GO) [55] terms associated with the retained Swiss-Prot homologs were obtained through species-independent UniProt-to-GO mappings and transferred to the corresponding *A. spinosa* protein models. GO terms were reconciled against the GO Basic ontology release dated 26 July 2026. Obsolete GO terms were remapped only when an explicit replaced_by relationship was provided; obsolete terms associated only with consider recommendations or lacking an explicit replacement were excluded from the current-term summaries. GO assignments were parsed and summarized using custom Python scripts according to the three principal GO domains: Biological Process, Molecular Function, and Cellular Component.

GO category counts were calculated as the number of protein-supported models associated with at least one term in each respective domain. Because individual protein models could receive multiple GO terms and could be represented in more than one GO domain, Biological Process, Molecular Function, and Cellular Component counts were treated as non-exclusive. GO-term frequencies were used to characterize functional representation among the annotated protein-supported models rather than as a measure of genome completeness, functional enrichment, or experimentally validated biological activity.

### 4.8. Identification and Comparative Analysis of Lipid Metabolism-Associated Homologs

Candidate homologs associated with fatty-acid and triacylglycerol metabolism were identified using a sequence-similarity-based approach with reference proteins from Arabidopsis thaliana and soybean (*Glycine max*). These species were selected because of the extensive annotation and functional characterization of proteins associated with plant fatty-acid metabolism and triacylglycerol (TAG) synthesis. Protein-supported Z-AL-IV-13 models were screened using BLAST- and DIAMOND-based sequence-similarity searches to identify candidates associated with fatty-acid synthesis, modification, glycerolipid metabolism, and TAG synthesis.

Targeted families included cytosolic and plastid acetyl-CoA carboxylase components, acyl carrier protein (ACP), malonyl-CoA:ACP transacylase (MCAT), ketoacyl-ACP synthases (KASI, KASII, and KASIII), 3-oxoacyl-ACP reductase (KAR), 3-hydroxyacyl-ACP dehydratase (HAD), enoyl-ACP reductase (ENR), stearoyl-ACP desaturase (SAD/SACPD), fatty-acid thioesterases (FATA, FATB, and TE3), fatty-acid desaturases, glycerol-3-phosphate acyltransferase (GPAT), lysophosphatidic acid acyltransferase (LPAT/LPAAT), phosphatidate phosphatase (PAH), diacylglycerol acyltransferase (DGAT), phospholipid:diacylglycerol acyltransferase (PDAT), lysophosphatidylcholine acyltransferase (LPCAT), and phosphatidylcholine:diacylglycerol cholinephosphotransferase (PDCT/ROD1).

Candidate assignments were evaluated using percentage identity, alignment length, bit score, E-value, alignment coverage, agreement with curated reference-protein annotations, and genomic coordinates. DIAMOND searches used an E-value threshold of 1 × 10^−5^, and the highest-scoring Swiss-Prot match was retained for the genome-wide similarity screen. For the targeted lipid analysis, candidates were not designated solely by a single universal percentage-identity or coverage cutoff; rather, sequence-similarity evidence was evaluated together with reference-family assignment and genomic context. Overlapping or fragmented protein-supported models mapping to the same genomic region were consolidated during locus-level interpretation to reduce inflation of candidate counts. Accordingly, the counts reported in Table 6 represent homology-supported assembly loci rather than raw protein-model counts or definitive biological gene-copy numbers. Sequence-based assignments were not interpreted as experimental confirmation of biochemical function or evidence of pathway completeness in *A. spinosa*.

For selected fatty-acid desaturase candidates, amino-acid sequences were aligned with reference FAD proteins using MAFFT v7.526 [56]. Alignments were trimmed using trimAl v1.5.rev1 [57], and phylogenetic relationships were inferred using FastTree v2.2.0 under the WAG amino-acid substitution model with the CAT approximation [34]. The resulting tree was midpoint-rooted for visualization. Placement of the *A. spinosa* candidates relative to characterized reference proteins was used as an additional line of evidence for sequence-based family classification, particularly for distinguishing FAD2-like from omega-3-desaturase-like candidates.

### 4.9. Repeat Identification and Genome Masking

Repetitive elements within the *A. spinosa* Z-AL-IV-13 assembly were characterized using a de novo repeat-discovery and masking approach. A de novo repeat library was generated from the assembly using RepeatModeler (v2.0.7) [58], which integrates repeat-identification algorithms including RECON [59] and RepeatScout [60] to identify and classify repetitive sequence families. The resulting assembly-derived consensus library contained 493 repeat consensus sequences and was subsequently used as a custom library with RepeatMasker (v4.2.3) [61]. RepeatMasker was rerun on the final contamination-screened Z-AL-IV-13 assembly (584,208,796 bp; 139,591 sequences) using RMBlast (v2.14.1+) as the search engine and otherwise default RepeatMasker settings.

RepeatMasker output was summarized to quantify the assembled sequence assigned to repeat categories and to calculate the proportion of the final assembly identified as repetitive. Because most detected interspersed repetitive sequence remained unclassified and repeat characterization was based on a fragmented short-read draft assembly and an assembly-derived de novo repeat consensus library, the resulting masked fraction was interpreted as a conservative estimate of recognizable repetitive sequence represented in the assembly rather than a definitive estimate of total repetitive content in the *A. spinosa* genome. Independent read-based estimates of repetitive sequence content were obtained from the GenomeScope2 analyses described in Section 4.3.

### 4.10. Bioinformatics Environment and Data Analysis

Computational analyses were performed using a combination of Amazon Web Services Elastic Compute Cloud (AWS EC2) resources and local computational environments. Software versions, principal parameters, and analytical procedures used for genome assembly, assembly-quality assessment, contamination screening, comparative genomics, repeat characterization, protein-supported annotation, functional annotation, and lipid-metabolism-associated homolog analysis are described in Section 4.1, Section 4.2, Section 4.3, Section 4.4, Section 4.5, Section 4.6, Section 4.7, Section 4.8 and Section 4.9.

Downstream data processing, statistical summaries, and figure generation were performed using custom Python scripts and Jupyter Notebook workflows. The computational environment included Python (v3.10.20), Jupyter Notebook (v7.6.1), pandas (v2.3.3), NumPy (v2.2.6), and matplotlib (v3.10.9). pandas and NumPy were used for data processing and numerical analyses, respectively, while matplotlib was used for data visualization. Custom shell scripts were additionally used for sequence-processing and annotation-summary tasks where indicated.

### 4.11. Reproducibility and Data Availability

Unless otherwise specified, bioinformatics analyses were performed using the parameters described in Section 4.1, Section 4.2, Section 4.3, Section 4.4, Section 4.5, Section 4.6, Section 4.7, Section 4.8, Section 4.9 and Section 4.10. Software versions and principal analysis settings are reported throughout the Methods to facilitate reproducibility. Custom scripts used for sequence processing, comparative analyses, statistical summaries, and figure generation are available from the corresponding author upon reasonable request.

The *A. spinosa* Z-AL-IV-13 genome assembly and associated project metadata have been deposited in GenBank under accession JCADCR000000000 (version JCADCR010000000), BioProject PRJNA1458287, and BioSample SAMN57501920.

## 5. Conclusions

This study presents a contamination-screened, genotype-specific draft genome resource for *Argania spinosa* Z-AL-IV-13 generated from high-coverage Illumina short-read sequencing. The final assembly comprises 584.21 Mb across 139,591 sequences but remains substantially fragmented and shows incomplete conserved gene-space recovery relative to chromosome-scale *A. spinosa* references. Independent BUSCO approaches produced similar completeness estimates of approximately 59%, while k-mer analyses estimated a larger haploid genome size of approximately 0.79–0.80 Gb. These results define the assembly as an incomplete draft rather than an alternative chromosome-scale reference.

Comparative genomic analyses nevertheless showed high sequence correspondence between regions represented in Z-AL-IV-13 and five independently generated *A. spinosa* assemblies. RepeatMasker identified 37.73% of the final assembly as repetitive, predominantly as unclassified interspersed repeats, whereas GenomeScope2 estimated a larger repetitive component of approximately 50–55%. Together, these results indicate that the assembly-based repeat fraction is conservative and that repetitive sequence likely contributed to the limited recovery and contiguity achievable with short reads.

Protein-supported annotation identified 44,777 transcript models and 172,399 CDS features, with 37,507 models producing significant Swiss-Prot matches. Targeted analyses further identified homology-supported assembly loci associated with multiple components of fatty-acid and triacylglycerol metabolism, including ACCase components, KAS, SACPD, FAD2, GPAT, LPAT/LPAAT, DGAT, PDAT, and acyl-ACP thioesterase families. These findings provide candidate sequences for future investigation of argan lipid metabolism but do not establish pathway completeness, definitive gene-copy numbers, expression, enzymatic activity, or specific contributions to argan oil composition.

Overall, Z-AL-IV-13 provides an independently generated genomic resource that complements recently available chromosome-scale *A. spinosa* assemblies by adding sequence information from an additional genotype. Its principal value lies in comparative sequence analysis, characterization of represented repetitive and protein-coding regions, and generation of candidate loci for subsequent functional investigation. Future integration of long-read sequencing, chromosome-scale scaffolding, transcriptomic evidence, and experimental functional analyses will be necessary to improve structural resolution, refine gene models, and determine the biological roles of candidate lipid-metabolism-associated genes.

## Figures and Tables

**Figure 1:**
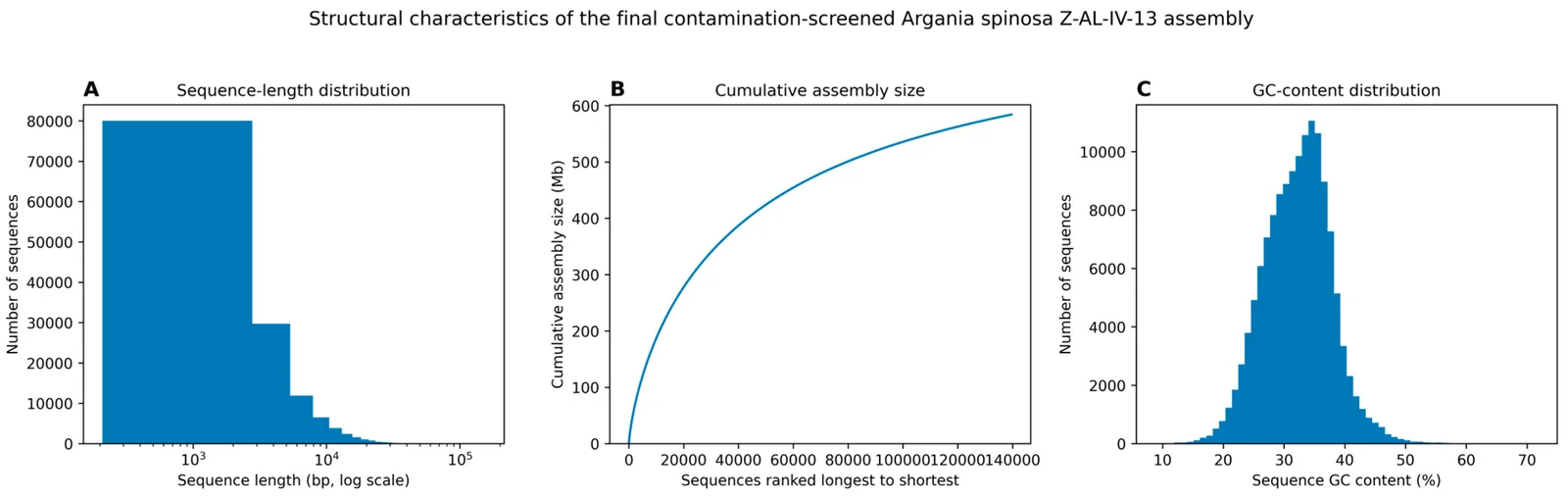
Structural characteristics of the final contamination-screened *A. spinosa* Z-AL-IV-13 genome assembly. (**A**) Distribution of sequence lengths displayed on a logarithmic scale. (**B**) Cumulative assembly size plotted across sequences ranked from longest to shortest, illustrating their contribution to the final 584.21-Mb assembly. (**C**) Distribution of sequence-level GC content. The final assembly comprises 139,591 sequences with an overall GC content of 32.35%.

**Figure 2:**
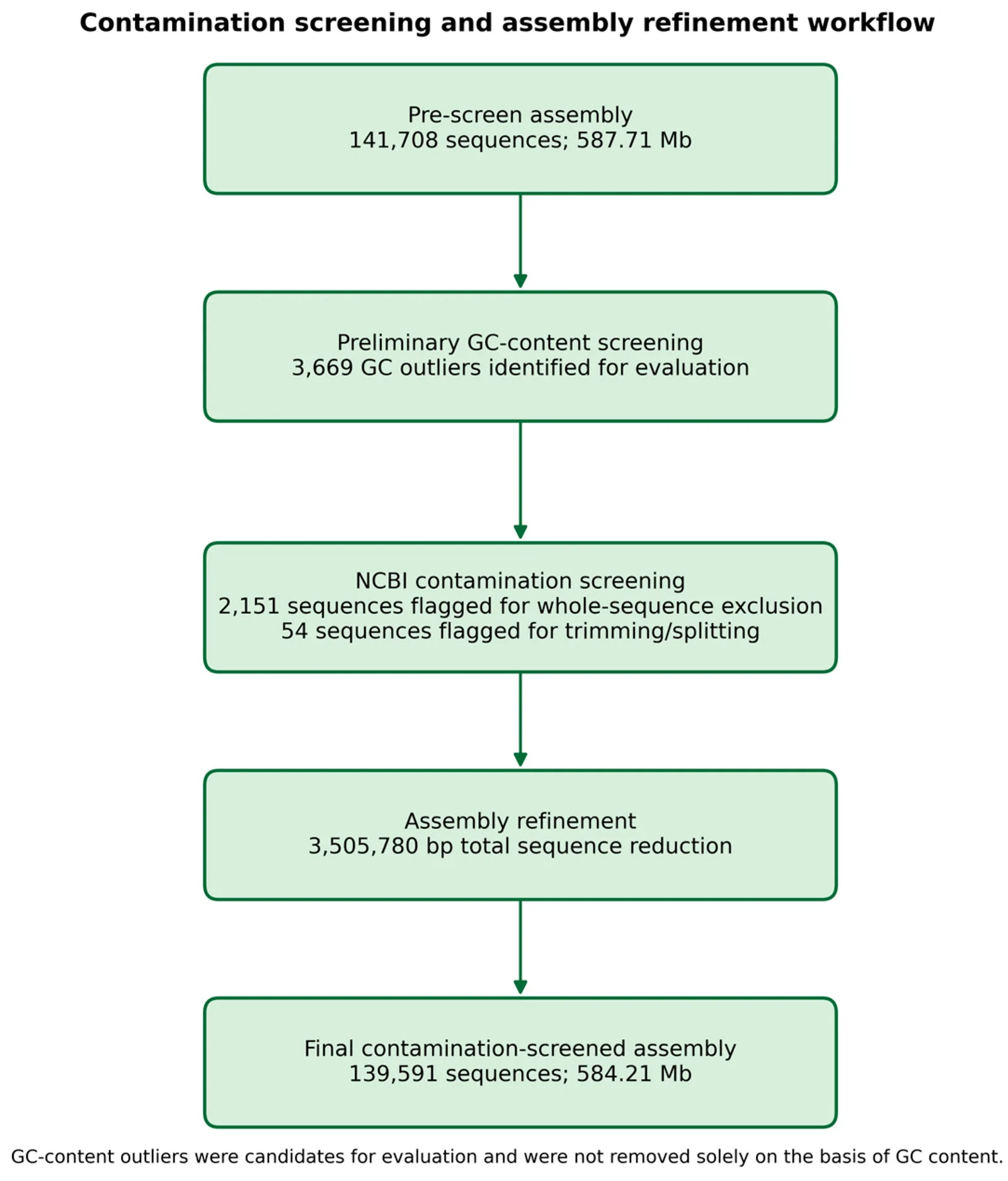
Workflow for contamination screening and refinement of the *Argania spinosa* Z-AL-IV-13 genome assembly. Preliminary GC-content screening identified sequences with atypical nucleotide composition for further evaluation but was not used alone as evidence of contamination. The pre-screen assembly was subsequently evaluated using the NCBI contamination-screening workflow, which flagged sequences for whole-sequence exclusion and trimming or splitting. Application of the NCBI screening recommendations reduced the assembly from 141,708 sequences (587.71 Mb) to a final contamination-screened assembly of 139,591 sequences (584.21 Mb). The final assembly was used for the revised downstream assembly-quality, comparative genomic, repeat, annotation, and functional analyses.

**Figure 3:**
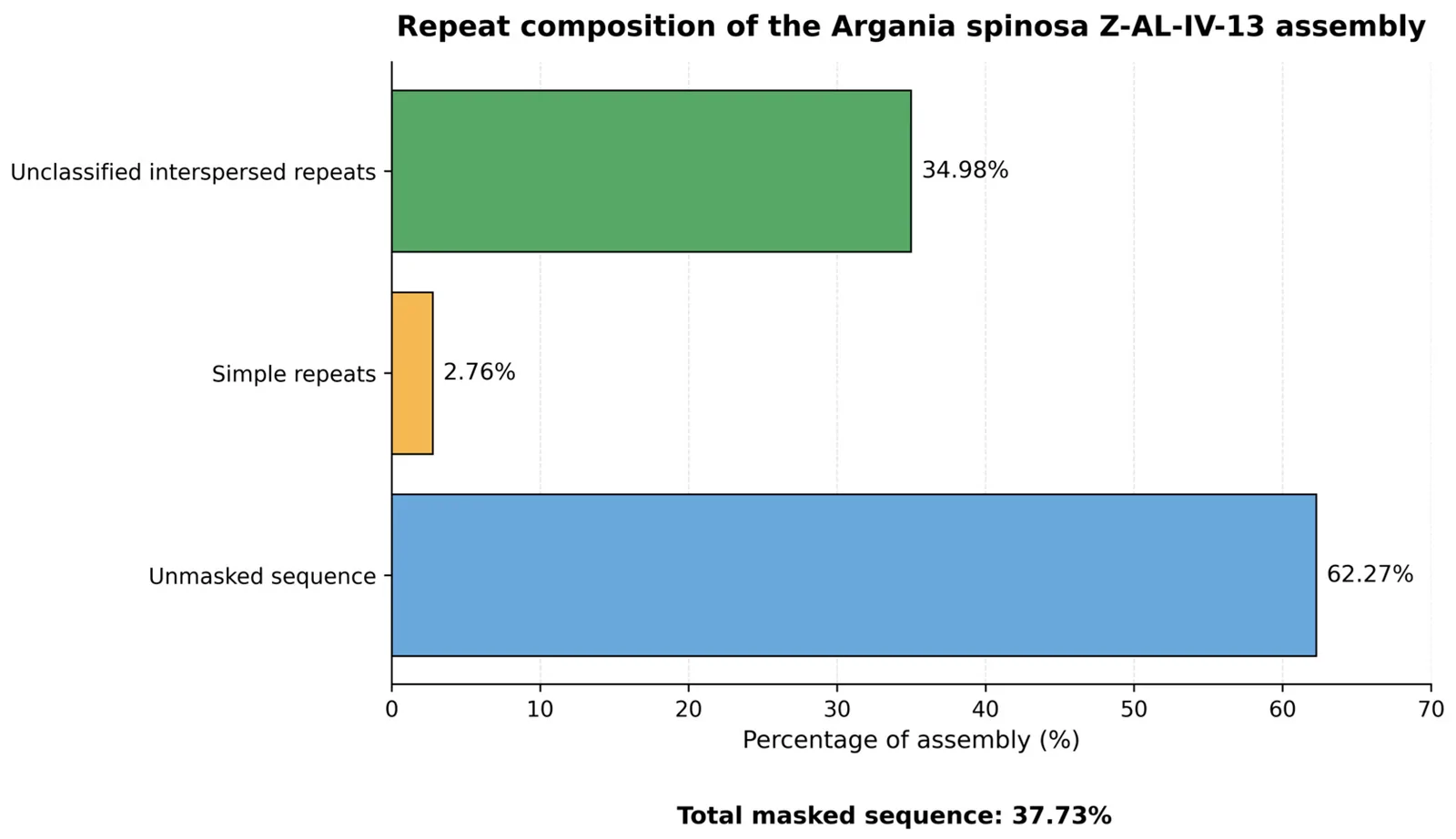
Repeat composition of the final contamination-screened *Argania spinosa* Z-AL-IV-13 assembly identified using an assembly-derived de novo repeat consensus library and RepeatMasker. Unclassified interspersed repeats accounted for 34.98% of the assembly and simple repeats for 2.76%, resulting in 37.73% total masked sequence. The remaining 62.27% was unmasked under the applied library and analysis conditions. Because most interspersed repeats remained unclassified, the masked fraction represents recognizable repetitive sequence in the draft assembly and should not be interpreted as a comprehensive estimate of total genome repetitiveness.

**Figure 4:**
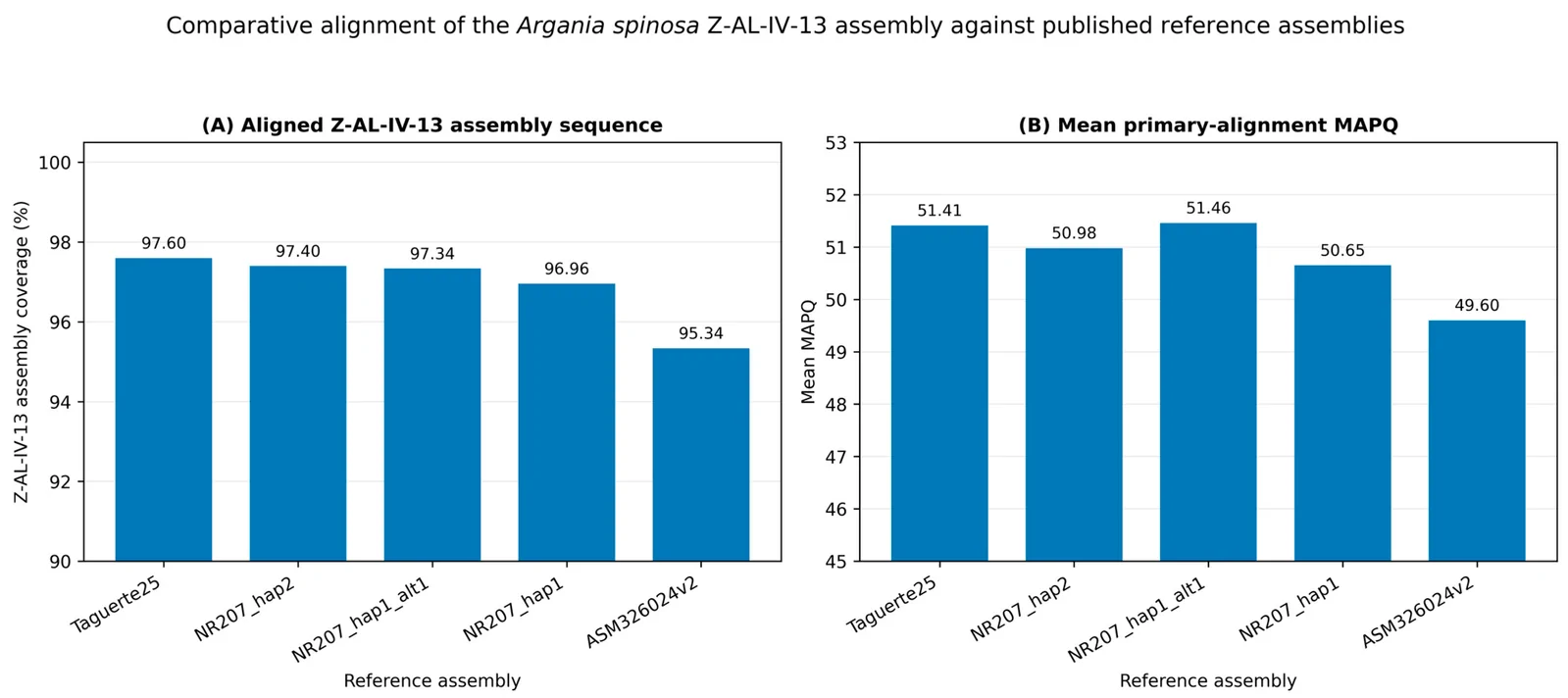
Comparative whole-genome alignment of the final contamination-screened *Argania spinosa* Z-AL-IV-13 assembly against five published *A. spinosa* reference assemblies. (**A**) Percentage of the Z-AL-IV-13 assembly represented by merged non-overlapping query intervals aligned to each reference. (**B**) Mean mapping quality (MAPQ) of primary minimap2 alignments. Alignment coverage represents correspondence of sequence present in the Z-AL-IV-13 assembly and should not be interpreted as an estimate of genome completeness.

**Figure 5:**
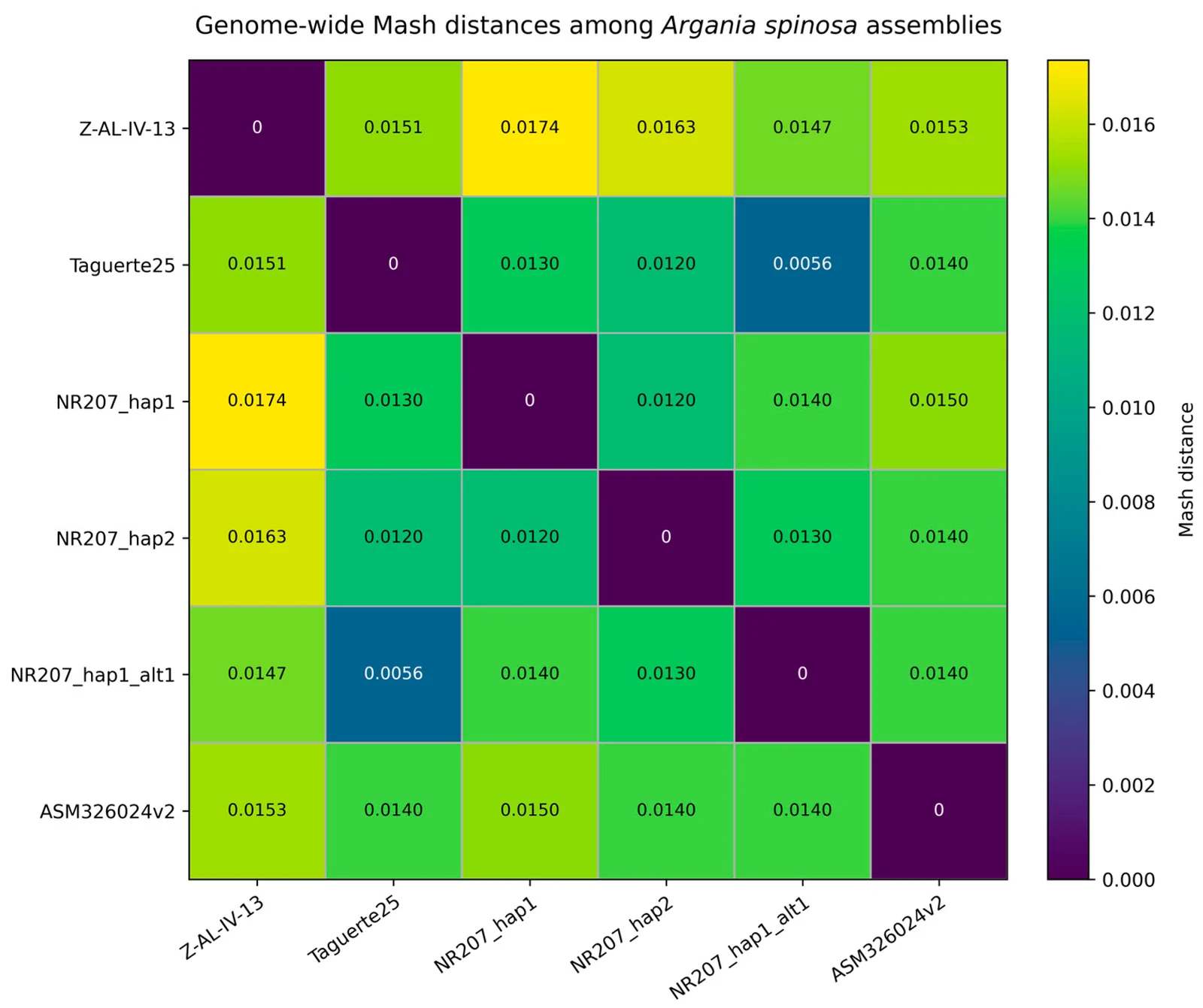
Genome-wide Mash distance heatmap comparing the *Argania spinosa* Z-AL-IV-13 assembly with five published *A. spinosa* reference assemblies: Taguerte25, NR207_hap1, NR207_hap2, NR207_hap1_alt1, and ASM326024v2. Pairwise Mash distances were calculated from whole-genome MinHash sketches. Lower Mash distances indicate greater overall sequence similarity between assemblies. Across all six assemblies, pairwise Mash distances ranged from 0.0056 to 0.0174.

**Figure 6:**
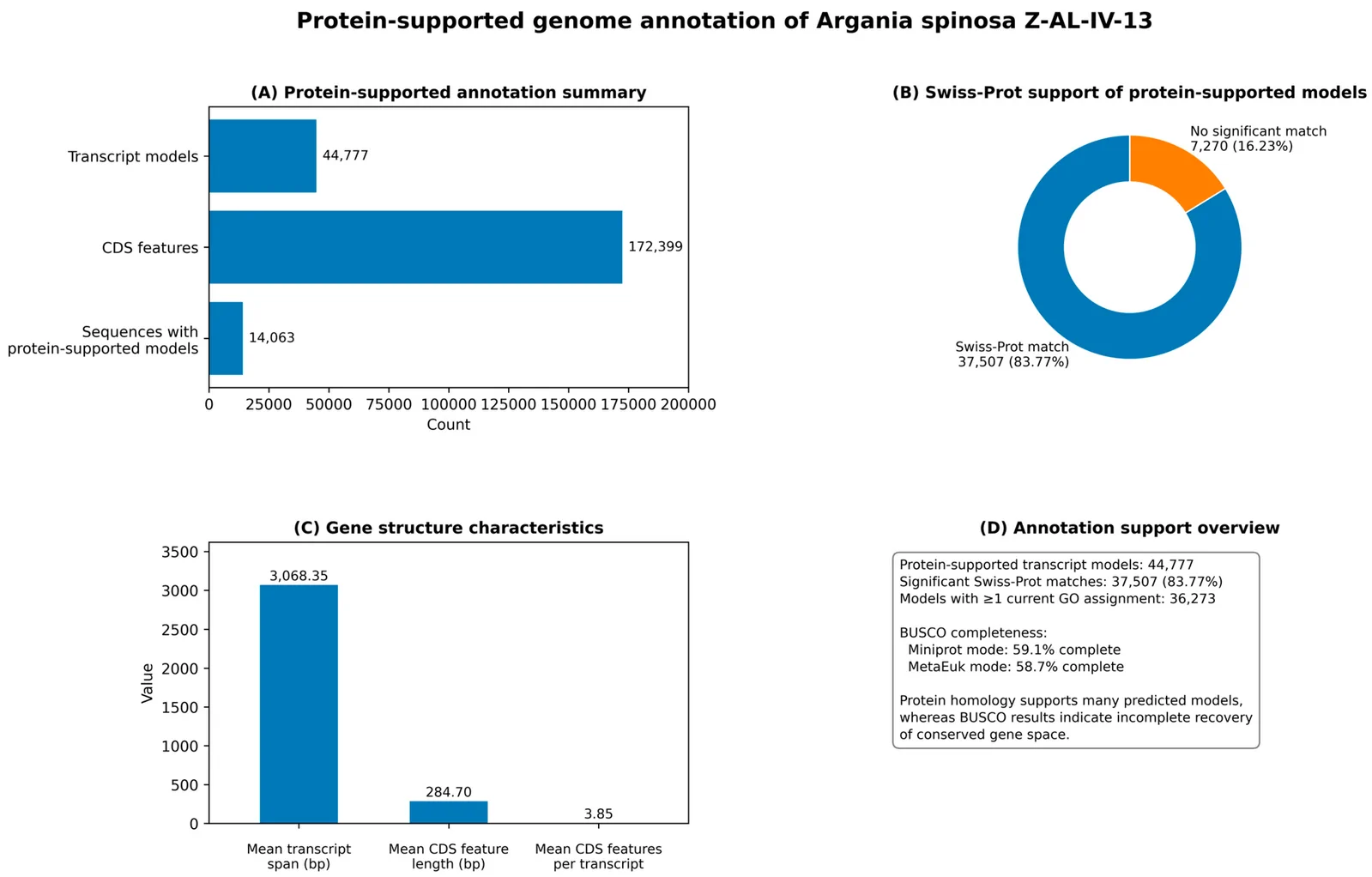
Protein-supported structural and functional annotation of the final contamination-screened *A. spinosa* Z-AL-IV-13 draft assembly. (**A**) Structural annotation summary showing the numbers of protein-supported transcript models, CDS features, and assembly sequences containing protein-supported models identified using miniprot with the Arabidopsis thaliana TAIR10 reference proteome. (**B**) Swiss-Prot similarity support among the 44,777 protein-supported models; 37,507 models (83.77%) produced a significant Swiss-Prot match using DIAMOND (E-value ≤ 1 × 10^−5^). (**C**) Structural characteristics of the protein-supported models, including mean transcript span, mean CDS feature length, and mean number of CDS features per transcript. (**D**) Summary of complementary annotation evidence, including Swiss-Prot similarity, Gene Ontology mapping, and BUSCO completeness. Protein-homology support should not be interpreted as evidence of complete gene-space recovery, as BUSCO analyses recovered approximately 59% of the conserved eudicot ortholog set as complete.

**Figure 7:**
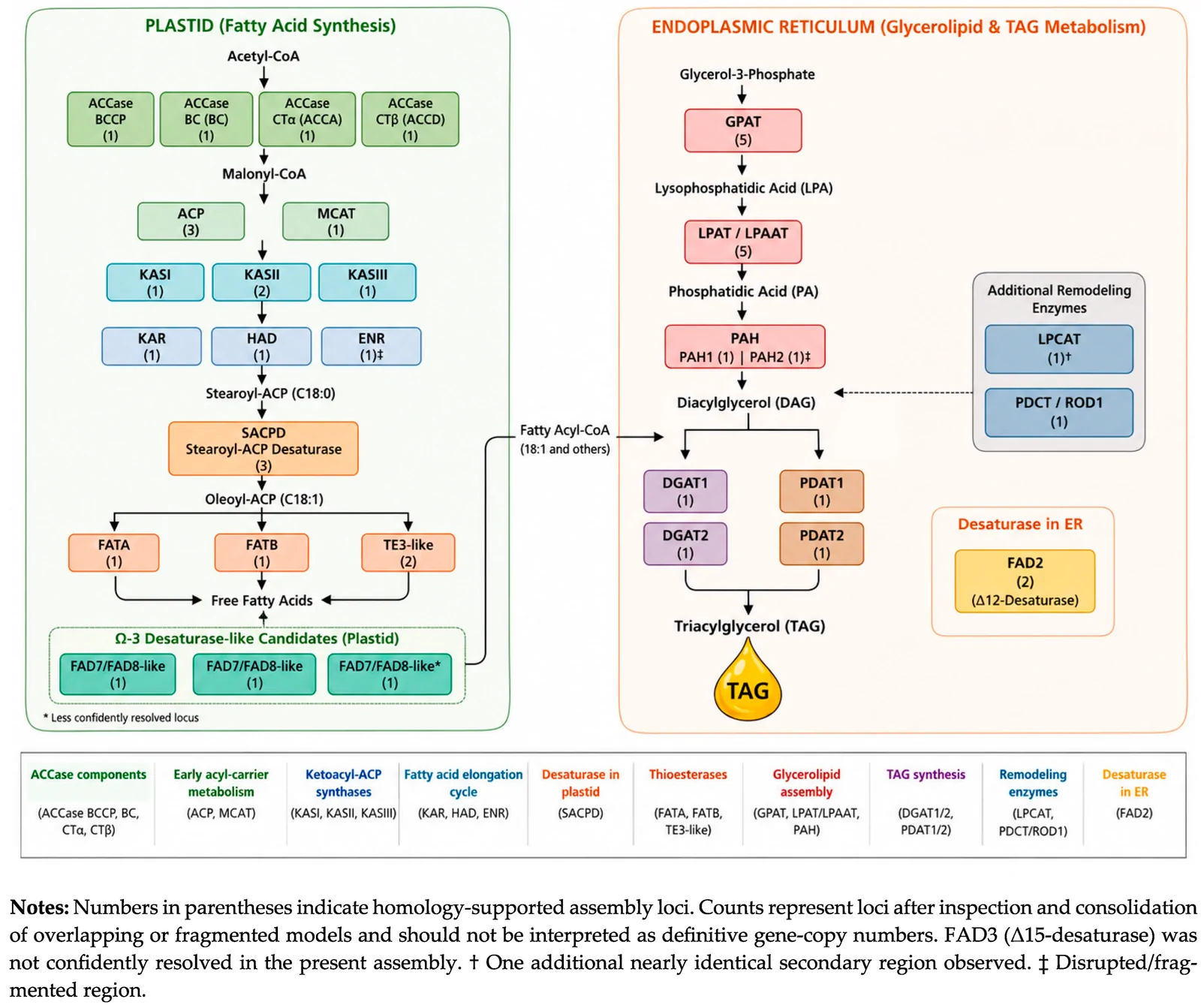
Schematic representation of lipid-metabolism-associated homologs identified in the Z-AL-IV-13 draft assembly. Homology-supported assembly loci were identified for multiple components associated with plastid fatty-acid synthesis and downstream glycerolipid and triacylglycerol metabolism. The diagram summarizes the metabolic context of the identified candidate families rather than demonstrating pathway completeness or enzymatic activity. Family assignments are based on sequence homology and, for selected fatty-acid desaturases, comparative phylogenetic analysis. Two FAD2-like loci and three omega-3-desaturase-like loci favoring FAD7/FAD8 classification were identified, whereas a distinct ER-associated FAD3 locus was not confidently resolved. Detailed locus counts and assignment qualifications are provided in Table 6.

**Figure 8:**
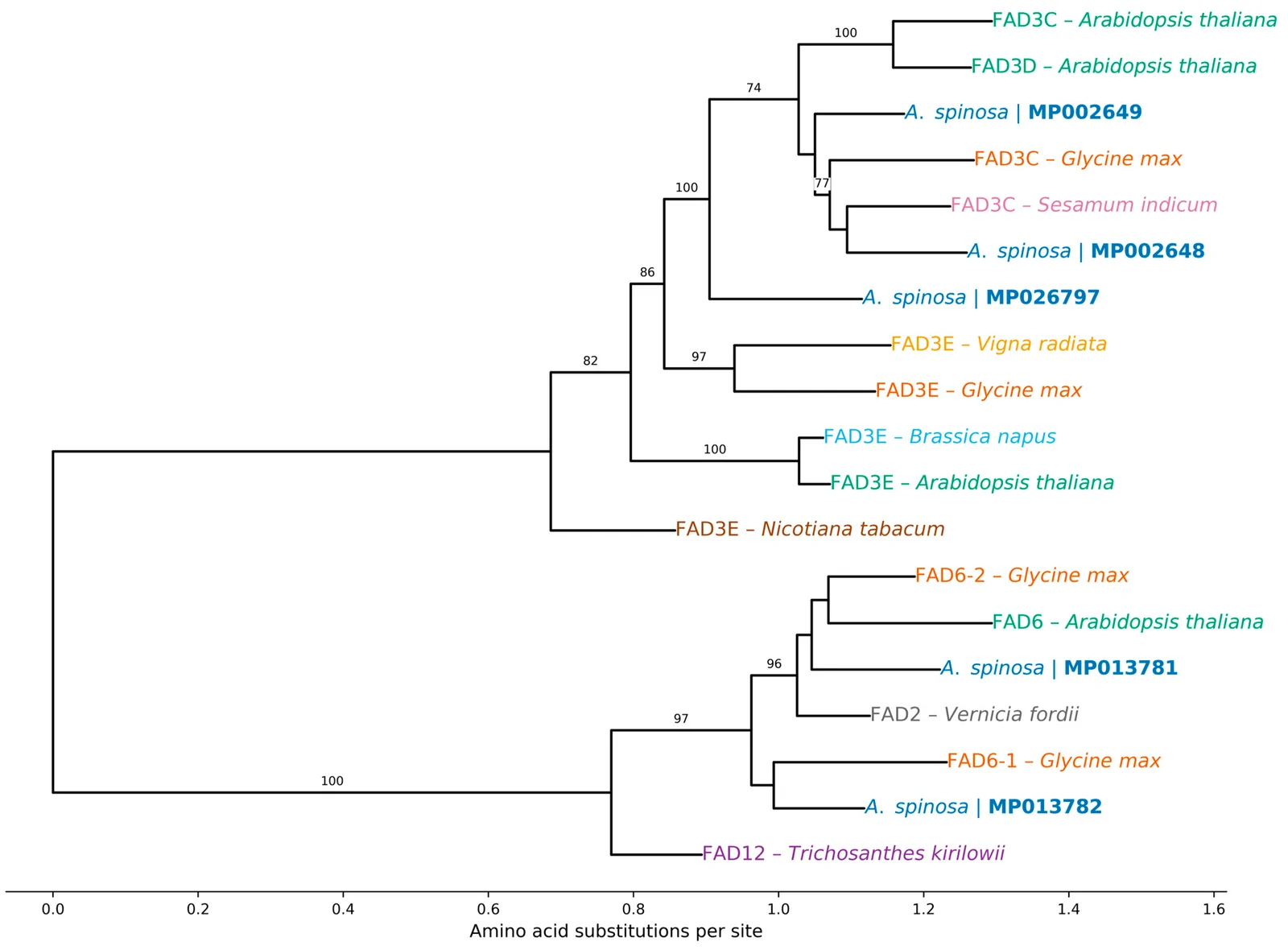
Phylogenetic classification of selected *A. spinosa* fatty acid desaturase homologs. Amino acid sequences of selected Z-AL-IV-13 fatty acid desaturase candidates and characterized reference desaturases were aligned using MAFFT v7.526 and trimmed using trimAl v1.5.rev1. Phylogenetic relationships were inferred using FastTree v2.2.0 under the WAG amino acid substitution model with the CAT approximation [34], and the tree was midpoint-rooted for visualization. Comparative placement supported the classification of two *A. spinosa* loci as FAD2-like and favored FAD7/FAD8-like assignments for three omega-3-desaturase-like loci, although one of the latter was less confidently resolved. A distinct ER-associated FAD3 locus was not confidently resolved. Branch lengths represent estimated amino acid substitutions per site. Phylogenetic placement was used as supporting evidence for sequence-based family classification and does not by itself establish biochemical function.

**Figure 9:**
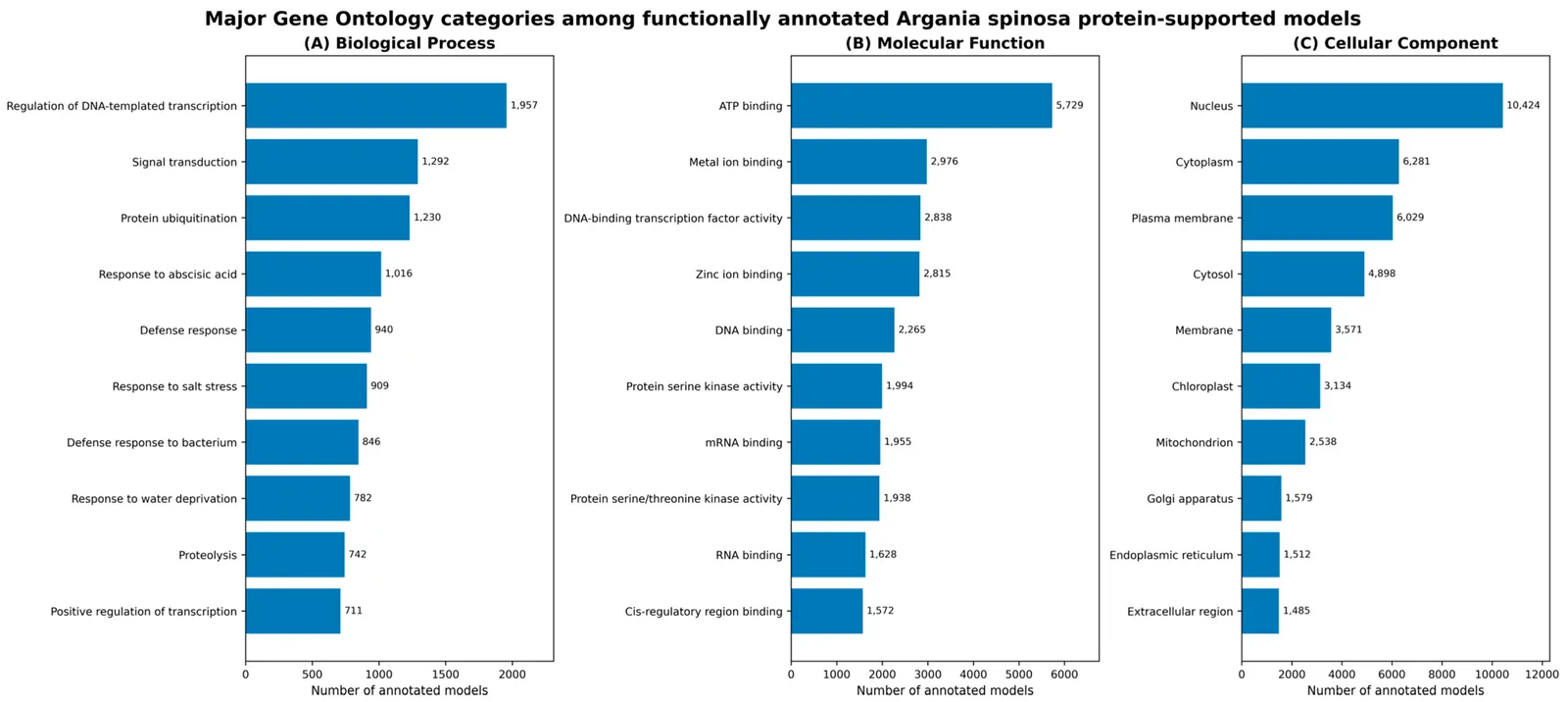
Major Gene Ontology (GO) terms associated with functionally annotated *A. spinosa* Z-AL-IV-13 protein-supported models. GO assignments were derived from Swiss-Prot best-hit associations and mapped to current Gene Ontology terms. The figure shows the ten most frequently represented terms within (**A**) Biological Process, (**B**) Molecular Function, and (**C**) Cellular Component categories. Counts represent protein-supported models associated with each GO term; individual models may contribute to multiple GO terms and namespaces. GO assignments describe functional representation among homology-supported models and should not be interpreted as evidence of complete gene-space recovery or pathway activity.

**Table 1:** Assembly statistics and completeness metrics for the final contamination-screened *A. spinosa* Z-AL-IV-13 genome assembly generated from Illumina short-read data. Metrics include assembly size, contiguity, GC content, BUSCO-based assessment of conserved gene-space recovery, GenomeScope2 genome-size and heterozygosity estimates, and solid read-derived 21-mer recovery.

Assembly Metric	Z-AL-IV-13
Assembly size (bp)	584,208,796
Number of sequences	139,591
Largest sequence (bp)	154,238
N50 (bp)	6841
L50	22,008
N90 (bp)	1639
L90	93,702
GC content (%)	32.35
Ns per 100 kbp	0
BUSCO—Miniprot	C: 59.1% [S: 51.4%, D: 7.7%], F: 25.4%, M: 15.6%
BUSCO—MetaEuk	C: 58.7% [S: 50.2%, D: 8.6%], F: 24.4%, M: 16.9%
GenomeScope2 genome size, k = 21	783–789 Mb
GenomeScope2 genome size, k = 31	795–801 Mb
GenomeScope2 heterozygosity, k = 21	1.57–1.63%
GenomeScope2 heterozygosity, k = 31	1.39–1.44%
Solid 21-mer recovery (≥20×)	79.32%

Note: Assembly statistics refer to the final NCBI contamination-screened assembly. BUSCO v6.0.0 analyses used the eudicots_odb10 lineage dataset (n = 2326) with Miniprot and MetaEuk as independent gene predictors. C, complete; S, complete and single-copy; D, complete and duplicated; F, fragmented; M, missing. GenomeScope2 estimates were obtained independently at k = 21 and k = 31. Solid 21-mer recovery represents the proportion of read-derived 21-mers with abundance ≥20× detected in the final assembly.

**Table 2:** Summary of GC-content screening and NCBI contamination-based refinement of the *A. spinosa* Z-AL-IV-13 assembly.

Screening/Refinement Metric	Result
**Preliminary GC-content screening**	**–**
GC-content screening interval	25–45%
Sequences outside GC interval	3669
Total length of GC outliers	6,214,397 bp
Fraction of pre-screen assembly	1.06%
GC outliers >5 kb	39
Total length of GC outliers >5 kb	270,755 bp
**NCBI contamination screening**	**–**
Pre-screen assembly	141,708 sequences; 587,714,576 bp
Sequences flagged for whole-sequence exclusion	2151
Total length of whole-sequence exclusions	3,498,876 bp
Fraction flagged for whole-sequence exclusion	0.595%
Sequences flagged for trimming/splitting	54
**Final assembly refinement**	**–**
Total sequence reduction	3,505,780 bp (0.597%)
Final contamination-screened assembly	139,591 sequences; 584,208,796 bp

Note: GC-content outliers were identified for further evaluation and were not considered contaminants solely on the basis of GC content. NCBI screening values refer to sequences flagged by the NCBI contamination-screening workflow. The total sequence reduction includes whole-sequence exclusions as well as sequence removed during trimming/splitting operations.

**Table 3:** RepeatMasker summary for the final contamination-screened Z-AL-IV-13 assembly using the assembly-derived de novo repeat consensus library.

Repeat Category	Number of Elements	Occupied Sequence (bp)	Percentage of Assembly (%)
Retroelements	0	0	0
DNA transposons	0	0	0
Rolling-circle elements	0	0	0
Unclassified interspersed repeats	762,070	204,335,864	34.98
Total interspersed repeats	—	204,335,864	34.98
Simple repeats	431,838	16,101,722	2.76
Low-complexity sequence	0	0	0
**Total masked sequence**	—	**220,437,586**	**37.73**

Note: RepeatMasker v4.2.3 was run with RMBlast v2.14.1+ against an assembly-derived de novo repeat consensus library containing 493 consensus sequences. Because the interspersed repeat fraction remained unclassified, the reported 37.73% represents recognizable repetitive sequence under the present library and assembly conditions and should be regarded as a conservative estimate rather than a comprehensive classification of the repetitive fraction. The absence of classified retroelements, DNA transposons, or rolling-circle elements in the RepeatMasker output should not be interpreted as evidence of their biological absence from the *A. spinosa* genome.

**Table 4:** Comparative whole-genome alignment statistics between the final contamination-screened *A. spinosa* Z-AL-IV-13 assembly and previously published *A. spinosa* reference genomes. Assembly coverage represents merged non-overlapping query intervals relative to the Z-AL-IV-13 assembly. Mean mapping quality (MAPQ) was calculated from primary minimap2 alignments (tp:A:P).

Reference	Z-AL-IV-13 Assembly Coverage (%)	Mean Primary-Alignment MAPQ
Taguerte25	97.60	51.41
NR207_hap2	97.40	50.98
hap1_alt1	97.34	51.46
NR207_hap1	96.96	50.65
ASM326024v2	95.34	49.60

**Table 5:** Protein-supported genome annotation statistics for the final contamination-screened *A. spinosa* Z-AL-IV-13 assembly based on miniprot alignment of Arabidopsis thaliana TAIR10 protein sequences. Metrics summarize protein-supported transcript models, coding sequence (CDS) features, assembly sequences containing protein-supported models, and structural characteristics of the resulting models.

Metric	Value
Protein-supported transcript models	44,777
CDS features	172,399
Assembly sequences with protein-supported models	14,063
Mean transcript span	3068.35 bp
Mean CDS feature length	284.70 bp
Mean CDS features per transcript	3.85

**Table 6:** Homology-supported assembly loci associated with fatty-acid and triacylglycerol metabolism in the *A. spinosa* Z-AL-IV-13 draft assembly. Locus counts were determined after inspection of protein-supported models and genomic coordinates to reduce inflation from fragmented or overlapping models. Counts represent assembly loci supported by sequence homology and should not be interpreted as definitive biological gene-copy numbers or evidence of pathway completeness.

Gene Family/Component	Homology-Supported Loci	Assignment/Interpretation
Cytosolic acetyl-CoA carboxylase (ACC1)	1	ACC1-like
Plastid heteromeric ACCase—BCCP	1	BCCP-family; additional weaker secondary fragment observed
Plastid heteromeric ACCase—biotin carboxylase (BC)	1	ACCC/BC-like
Plastid heteromeric ACCase—carboxyltransferase α (CTα)	1	CAC3/ACCA-like
Plastid heteromeric ACCase—carboxyltransferase β (CTβ)	1	ACCD-like
Acyl carrier protein (ACP)	3	Chloroplast ACP-family loci
Malonyl-CoA:ACP transacylase (MCAT)	1	MCAT-like
Ketoacyl-ACP synthase I (KASI)	1	KASI-like
Ketoacyl-ACP synthase II (KASII)	2	KASII-like
Ketoacyl-ACP synthase III (KASIII)	1	KASIII-like
3-Oxoacyl-ACP reductase (KAR)	1	KAR-like
3-Hydroxyacyl-ACP dehydratase (HAD)	1	HAD-like
Enoyl-ACP reductase (ENR)	1	ENR/MOD1-like; additional highly similar secondary region observed
Stearoyl-ACP desaturase (SACPD)	3	SACPD-like assembly loci/regions
Acyl-ACP thioesterase A (FATA)	1	FATA-like
Acyl-ACP thioesterase B (FATB)	1	FATB-like
Acyl-ACP thioesterase-like (TE3)	2	TE3-like
Fatty acid desaturase 2 (FAD2)	2	Distinct FAD2-like loci
Omega-3 fatty acid desaturase	3	FAD7/FAD8-favored loci; one less confidently resolved
Glycerol-3-phosphate acyltransferase (GPAT)	5	GPAT-family loci
Lysophosphatidic acid acyltransferase (LPAT/LPAAT)	5	LPAT/LPAAT-family loci
Phosphatidate phosphatase (PAH1)	1	PAH1-like
Phosphatidate phosphatase (PAH2)	1	PAH2-like; disrupted/fragmented region
Diacylglycerol acyltransferase 1 (DGAT1)	1	DGAT1-like
Diacylglycerol acyltransferase 2 (DGAT2)	1	DGAT2-like
Phospholipid:diacylglycerol acyltransferase 1 (PDAT1)	1	PDAT1-like
Phospholipid:diacylglycerol acyltransferase 2 (PDAT2)	1	PDAT2-like
Lysophosphatidylcholine acyltransferase (LPCAT)	1	LPCAT-family locus; additional nearly identical secondary region observed
Phosphatidylcholine:diacylglycerol cholinephosphotransferase (PDCT/ROD1)	1	ROD1/PDCT-like
Glycerol-3-phosphate dehydrogenase, GPDHp-like	1	NAD-dependent GPDH-like
Glycerol-3-phosphate dehydrogenase, GLY1-like	1	NAD-dependent GPDH-like

## Data Availability

The Whole-Genome Shotgun project has been deposited in DDBJ/ENA/GenBank under the accession JCADCR000000000. The version described in this paper is JCADCR010000000. The project is associated with BioProject PRJNA1458287 and BioSample SAMN57501920.

## Supplementary Materials

The following supporting information can be downloaded at https://www.mdpi.com/article/10.3390/plants15192945/s1. Figure S1: Preliminary GC-content screening of sequences in the Argania spinosa Z-AL-IV-13 draft assembly prior to final NCBI contamination screening. Sequence GC content is plotted against sequence length on a logarithmic scale. Dashed red lines indicate the preliminary GC-content thresholds of 25% and 45% used to identify candidate outlier sequences for further evaluation. GC-content outliers were not considered contaminants solely on the basis of GC content. Final sequence exclusion and trimming were determined subsequently using the NCBI contamination-screening results; Table S1: Pairwise Mash distances among the final contamination-screened Argania spinosa Z-AL-IV-13 assembly and published A. spinosa reference genomes. Mash distances were calculated using Mash v2.3 from whole-genome MinHash sketch comparisons and provide an alignment-independent measure of overall sequence similarity between assemblies. Lower Mash distance values indicate greater sequence similarity; Table S2: Homology-supported assembly loci associated with fatty-acid and triacylglycerol metabolism in the Argania spinosa Z-AL-IV-13 draft genome assembly. Candidate loci were identified through sequence-similarity searches against characterized plant lipid-metabolism-associated proteins and consolidated at the assembly-locus level after consideration of reference assignment, alignment evidence, and assembly context. Multiple protein-supported models or reference-protein hits associated with the same assembly region were not interpreted as independent gene copies. Counts therefore represent homology-supported assembly loci or regions and should not be interpreted as definitive biological gene-copy numbers or evidence of pathway completeness; Table S3: Most frequent Gene Ontology (GO) terms associated with functionally annotated Argania spinosa Z-AL-IV-13 protein-supported models. GO assignments were inherited from Swiss-Prot best-hit associations and mapped to current terms in the GO Basic ontology (release 26 July 2026). The 15 most frequent terms are reported separately for biological process (BP), molecular function (MF), and cellular component (CC). Counts represent protein-supported models associated with each GO term. Individual models may have multiple GO assignments within or across namespaces; therefore, counts are non-exclusive and should not be summed to estimate the total number of functionally annotated models; Table S4: Assembly statistics for the final contamination-screened Argania spinosa Z-AL-IV-13 draft genome assembly. Statistics were calculated directly from the final assembly FASTA after sequence exclusion and trimming based on NCBI contamination screening.

## Abbreviations

The following abbreviations are used in this manuscript:

ACC	Acetyl-CoA Carboxylase
BUSCO	Benchmarking Universal Single-Copy Orthologs
DGAT	Diacylglycerol acyltransferase
FAD	Fatty Acid Desaturase
GPAT	Glycerol-3-phosphate Acyltransferase
KAS	Ketoacyl Synthase
MAPQ	Mapping Quality
TAG	Triacylglycerol
