# Supplementary material for: A Draft Genome Assembly of Argania spinosa Provides Insights into Lipid Metabolism-Associated Genes and Repeat-Rich Genome Architecture
Source: Plants (Basel). 2026 Sep 26;15(19):2945. doi: 10.3390/plants15192945 (PMC13667010; doi:10.3390/plants15192945)
Supplement: Supplementary file 1 [file plants-15-02945-s001.zip › plants-4503185-supplementary.pdf]

Supplementary Figures and Tables:

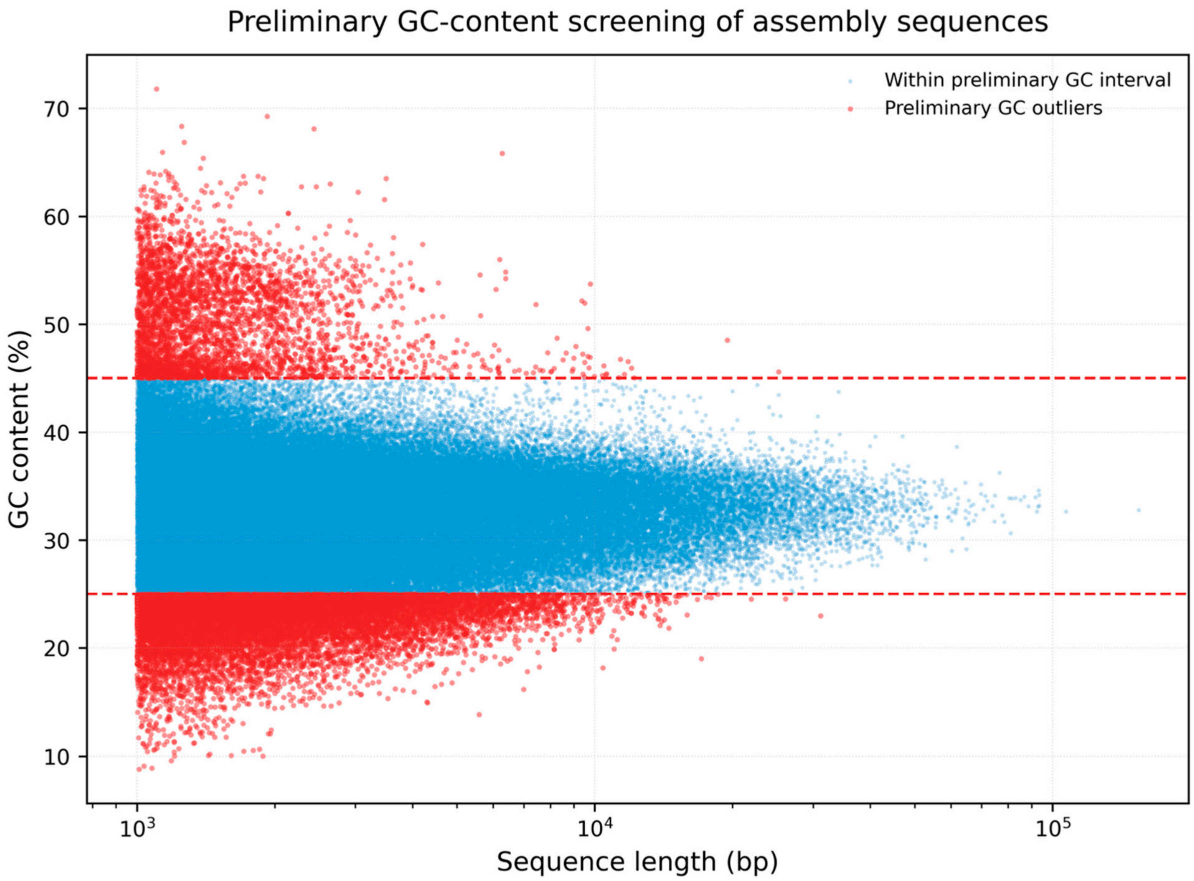

Figure S1. Preliminary GC-content screening of sequences in the Argania spinosa Z-AL-IV-13 draft assembly prior to final NCBI contamination screening. Sequence GC content is plotted against sequence length on a logarithmic scale. Dashed red lines indicate the preliminary GC-content thresholds of 25% and 45% used to identify candidate outlier sequences for further evaluation. GC-content outliers were not considered contaminants solely on the basis of GC content. Final sequence exclusion and trimming were determined subsequently using the NCBI contamination-screening results.

Table S1. Pairwise Mash distances among the final contamination-screened Argania spinosa Z-AL-IV-13 assembly and published A. spinosa reference genomes. Mash distances were calculated using Mash v2.3 from whole-genome MinHash sketch comparisons and provide an alignment-independent measure of overall sequence similarity between assemblies. Lower Mash distance values indicate greater sequence similarity.

Assembly 1	Assembly 2	Mash Distance
Z-AL-IV-13	Taguerte25	0.0150813
Z-AL-IV-13	NR207_hap1	0.017359
Z-AL-IV-13	NR207_hap2	0.0162749
Z-AL-IV-13	NR207_hap1_alt1	0.0146626
Z-AL-IV-13	ASM326024v2	0.015347

Taguerte25	NR207_hap1	0.0132573
Taguerte25	NR207_hap2	0.0123533
Taguerte25	NR207_hap1_alt1	0.00557568
Taguerte25	ASM326024v2	0.0138985
NR207_hap1	NR207_hap2	0.0124
NR207_hap1	NR207_hap1_alt1	0.0140996
NR207_hap1	ASM326024v2	0.0154006
NR207_hap2	NR207_hap1_alt1	0.0129677
NR207_hap2	ASM326024v2	0.0140996
NR207_hap1_alt1	ASM326024v2	0.0143537

Table S2. Homology-supported assembly loci associated with fatty-acid and triacylglycerol metabolism in the Argania spinosa Z-AL-IV-13 draft genome assembly. Candidate loci were identified through sequence-similarity searches against characterized plant lipid-metabolism-associated proteins and consolidated at the assembly-locus level after consideration of reference assignment, alignment evidence, and assembly context. Multiple protein-supported models or reference-protein hits associated with the same assembly region were not interpreted as independent gene copies. Counts therefore represent homology-supported assembly loci or regions and should not be interpreted as definitive biological gene-copy numbers or evidence of pathway completeness.

Family	Sequence-based assignment	Supported loci / regions	Evidence / interpretation
ACCase/ACC1	Cytosolic ACC1-like	1	Confident
ACCase/BCCP	Chloroplastic BCCP-family	1	Plus one weaker secondary fragment
ACCase/BC	Chloroplastic biotin carboxylase-like	1	Confident
ACCase/CTα	Chloroplastic CAC3/ACCA-like	1	Confident
ACCase/CTβ	Chloroplastic ACCD-like	1	Confident
ACP	Chloroplastic ACP-family	3	Assembly loci
MCAT	Malonyl-CoA:ACP transacylase-like	1	Confident
KASI	KASI-like	1	Confident
KASII	KASII-like	2	Assembly loci
KASIII	KASIII-like	1	Confident
KAR	3-Oxoacyl-ACP reductase-like	1	Confident
HAD	3-Hydroxyacyl-ACP dehydratase-like	1	Confident
ENR	Enoyl-ACP reductase/MOD1-like	1	Plus one highly similar secondary region
SACPD	Stearoyl-ACP desaturase-like	3	Assembly loci/regions
FATA	FATA-like	1	Confident
FATB	FATB-like	1	Confident
TE3	Acyl-ACP thioesterase-like	2	Assembly loci
FAD2	FAD2-like	2	Distinct loci
Omega-3 desaturase	FAD7/FAD8-favored omega-3-desaturase-like	3	One locus less confidently resolved
GPAT	GPAT-family	5	GPAT3/5/8/9 and ATS1/GPAT2-like

LPAT/LPAAT	LPAT/LPAAT-family	5	LPAT2/4/5, BAT2/LPAT, and LPAAT-like
PAH1	Phosphatidate phosphatase PAH1-like	1	Confident
PAH2	PAH2-like	1	Disrupted/fragmented region
DGAT1	DGAT1-like	1	Confident
DGAT2	DGAT2-like	1	Confident
PDAT1	PDAT1-like	1	Confident
PDAT2	PDAT2-like	1	Confident
LPCAT	LPCAT-family	1	Plus one nearly identical secondary region
PDCT/ROD1	ROD1/PDCT-like	1	Confident
GPDHp	GPDHp-like	1	Confident
GLY1	GLY1-like GPDH	1	Confident

Note: Locus counts summarize sequence-homology-supported assembly regions after consolidation of redundant or overlapping candidate models. Secondary fragments or highly similar assembly regions are indicated separately and were not counted as additional confident loci. Because the Z-AL-IV-13 assembly is fragmented and shows incomplete gene-space recovery, these values should not be interpreted as definitive biological gene-copy numbers. Failure to confidently resolve an additional family member should likewise not be interpreted as evidence of biological gene absence. Functional assignments are sequence based and do not establish expression or biochemical activity.

Table S3. Most frequent Gene Ontology (GO) terms associated with functionally annotated Argania spinosa Z-AL-IV-13 protein-supported models. GO assignments were inherited from Swiss-Prot best-hit associations and mapped to current terms in the GO Basic ontology (release 26 July 2026). The 15 most frequent terms are reported separately for biological process (BP), molecular function (MF), and cellular component (CC). Counts represent protein-supported models associated with each GO term. Individual models may have multiple GO assignments within or across namespaces; therefore, counts are non-exclusive and should not be summed to estimate the total number of functionally annotated models.

**Namespace**	**GO ID**	**GO term**	**Model count**
BP	GO:0006355	Regulation of DNA-templated transcription	1,957
BP	GO:0007165	Signal transduction	1,292
BP	GO:0016567	Protein ubiquitination	1,230
BP	GO:0009737	Response to abscisic acid	1,016
BP	GO:0006952	Defense response	940
BP	GO:0009651	Response to salt stress	909
BP	GO:0042742	Defense response to bacterium	846
BP	GO:0009414	Response to water deprivation	782
BP	GO:0006508	Proteolysis	742
BP	GO:0045893	Positive regulation of DNA-templated transcription	711
BP	GO:0006357	Regulation of transcription by RNA polymerase II	703
BP	GO:0015031	Protein transport	672
BP	GO:0009409	Response to cold	669
BP	GO:0006397	mRNA processing	665

BP	GO:0071555	Cell wall organization	651
MF	GO:0005524	ATP binding	5,729
MF	GO:0046872	Metal ion binding	2,976
MF	GO:0003700	DNA-binding transcription factor activity	2,838
MF	GO:0008270	Zinc ion binding	2,815
MF	GO:0003677	DNA binding	2,265
MF	GO:0106310	Protein serine kinase activity	1,994
MF	GO:0003729	mRNA binding	1,955
MF	GO:0004674	Protein serine/threonine kinase activity	1,938
MF	GO:0003723	RNA binding	1,628
MF	GO:0000976	Transcription cis-regulatory region binding	1,572
MF	GO:0016887	ATP hydrolysis activity	1,152
MF	GO:0061630	Ubiquitin protein ligase activity	818
MF	GO:0043565	Sequence-specific DNA binding	733
MF	GO:0003735	Structural constituent of ribosome	726
MF	GO:0005516	Calmodulin binding	687
CC	GO:0005634	Nucleus	10,424
CC	GO:0005737	Cytoplasm	6,281
CC	GO:0005886	Plasma membrane	6,029
CC	GO:0005829	Cytosol	4,898
CC	GO:0016020	Membrane	3,571
CC	GO:0009507	Chloroplast	3,134
CC	GO:0005739	Mitochondrion	2,538
CC	GO:0005794	Golgi apparatus	1,579
CC	GO:0005783	Endoplasmic reticulum	1,512
CC	GO:0005576	Extracellular region	1,485
CC	GO:0009506	Plasmodesma	1,446
CC	GO:0005789	Endoplasmic reticulum membrane	1,239
CC	GO:0009536	Plastid	1,219
CC	GO:0000139	Golgi membrane	1,047
CC	GO:0005730	Nucleolus	1,017

Note: Of the 37,507 protein-supported models with a Swiss-Prot best hit, 36,273 (96.71%) received at least one current GO assignment. Biological process, molecular function, and cellular component assignments were associated with 31,407, 31,833, and 33,013 models, respectively. These categories overlap because individual models may receive assignments in more than one GO namespace. GO terms were inherited from Swiss-Prot homolog associations; this analysis represents functional annotation rather than GO enrichment and does not establish experimentally validated functions in A. spinosa.

Table S4. Assembly statistics for the final contamination-screened Argania spinosa Z-AL-IV-13 draft genome assembly. Statistics were calculated directly from the final assembly FASTA after sequence exclusion and trimming based on NCBI contamination screening.

**Metric**	**Value**
Number of sequences	139,591
Total assembly length (bp)	584,208,796
Largest sequence (bp)	154,238
N50 (bp)	6,841
L50	22,008
N90 (bp)	1,639
L90	93,702
GC content (%)	32.35
Ns per 100 kbp	0
